# Newborn infant skin gene expression: Remarkable differences versus adults

**DOI:** 10.1371/journal.pone.0258554

**Published:** 2021-10-19

**Authors:** Marty O. Visscher, Ping Hu, Andrew N. Carr, Charles C. Bascom, Robert J. Isfort, Kellen Creswell, Rachel Adams, Jay P. Tiesman, Karen Lammers, Vivek Narendran

**Affiliations:** 1 Skin Sciences, Program, Cincinnati Children’s Hospital Medical Center, Cincinnati, OH, United States of America; 2 James L. Winkle College of Pharmacy, University of Cincinnati, Cincinnati, OH, United States of America; 3 The Procter & Gamble Company, Cincinnati, OH, United States of America; 4 Perinatal Institute, Cincinnati Children’s Hospital Medical Center, Cincinnati, OH, United States of America; INSERM, FRANCE

## Abstract

At birth, human infants are poised to survive in harsh, hostile conditions. An understanding of the state of newborn skin development and maturation is key to the maintenance of health, optimum response to injury, healing and disease. The observational study collected full-thickness newborn skin samples from 27 infants at surgery and compared them to skin samples from 43 adult sites protected from ultraviolet radiation exposure, as the standard for stable, mature skin. Transcriptomics profiling and gene set enrichment analysis were performed. Statistical analysis established over 25,000 differentially regulated probe sets, representing 10,647 distinct genes, in infant skin compared to adult skin. Gene set enrichment analysis showed a significant increase in 143 biological processes (adjusted p < 0.01) in infant skin, versus adult skin samples, including extracellular matrix (ECM) organization, cell adhesion, collagen fibril organization and fatty acid metabolic process. ECM organization and ECM structure organization were the biological processes in infant skin with the lowest adjusted P-value. Genes involving epidermal development, immune function, cell differentiation, and hair cycle were overexpressed in adults, representing 101 significantly enriched biological processes (adjusted p < 0.01). The processes with the highest significant difference were skin and epidermal development, e.g., keratinocyte differentiation, keratinization and cornification intermediate filament cytoskeleton organization and hair cycle. Enriched Gene Ontology (GO) biological processes also involved immune function, including antigen processing and presentation. When compared to ultraviolet radiation-protected adult skin, our results provide essential insight into infant skin and its ability to support the newborn’s preparedness to survive and flourish, despite the infant’s new environment laden with microbes, high oxygen tension and potential irritants. This fundamental knowledge is expected to guide strategies to protect and preserve the features of unperturbed, young skin.

## Introduction

Newborn infant skin is truly remarkable, characterized by its notable softness, smoothness and uniformity, yet poised to survive in harsh, hostile conditions following birth [1]. An understanding of this pristine infant skin is key to the maintenance of health, optimum response to insults and restoration of barrier damage and avoidance of disease, e.g., atopic dermatitis. In contrast, adult skin reflects the accumulation of years of assault from its environment, including ultraviolet radiation [2], endogenous aging, changes in temperature and humidity, exposure to irritants [3] and disease, to name a few. Exploration and understanding of the biological processes in newborn skin is a critical factor in mitigating these negative outcomes.

Full-term newborn skin is well-formed at birth with low transepidermal water loss (TEWL), equal to, or lower, than that of adults [4, 5]. The epidermis is thinner than that found in adults and rete ridges are not yet developed [6]. At birth, infants rapidly move from an aqueous, warm, often sterile uterine environment to cooler, dry, gaseous conditions. The transition is physiologically profound, perhaps the most dramatic life event. Provision of innate immunity is an essential skin function, particularly at birth. Innate immunity is conferred through a complex balance of structural proteins, lipids, Langerhans cells, pro- and anti-inflammatory cytokines and the physical stratum corneum (SC) barrier.

Newborn skin undergoes considerable changes after birth that continue beyond the first year of life. Skin hydration decreases rapidly on postnatal day 1 and then increases over the first month [7]. Notable dryness and scaling occur, owing, in part, to the low levels of water binding molecules, known as natural moisturizing factor (NMF) [8]. The skin pH of ~7 at birth decreases rapidly during days 1–4, decreases gradually as the acid mantle develops and is, thereafter, influenced by gestational age (GA) and time from birth [8]. An acidic pH is necessary for effective function of enzymes involved in SC formation and integrity, including lipid metabolism, bilayer structure formation, ceramide (SC lipid) synthesis and desquamation [9, 10]. Multiple mechanisms are in play, including (1) filaggrin proteolysis to amino acids, pyrrolidone carboxylic acid and urocanic acid; (2) phospholipid hydrolysis to FFA by secretory phospholipases; (3) acidification in the lower SC by a Na^+^H^+^ antiporter mechanism (NHE1); (4) dispersion of melanin granules to release H+; and (5) cholesterol sulfate reaction to produce H^+^ [11]. Human skin is the largest interface for microbiome interaction [12]. Skin surface microbiota respond to inflammation, contribute to immunity via modulation of IL1a [13] and regulate antimicrobial peptides (AMPs), e.g., β-defensins and cathelicidins [14, 15]. An acidic skin surface facilitates the activity of AMPs and increases microbiome diversity, including corynebacteria and staphylococci (commensals), but it obstructs pathogenic organisms [16]. The adult skin microbiome is impacted by properties linked to the epidermal barrier [17]. Since such properties change during newborn adaptation for full-term infants, dynamic alterations in the skin microbiome are expected [18].

In a previous report, we described neonatal skin barrier adaptation and functional integrity using targeted proteomic skin biomarker analysis from the outer SC [19]. At birth, neonatal skin showed upregulation of processes allowed by the lower pH of infant skin. These include the production of water-binding natural moisturizer (NMF), prevention of protease-based desquamation and augmentation of skin barrier antimicrobial function. These processes can be seen in the markedly different array of protein biomarkers shortly after birth and 2–3 months later in comparison to stable adult skin. Neonatal skin exhibited adaptive changes over time, presumably to provide innate immunity and continue barrier development. The present work aimed to extend the comparison by examining gene expression in full-thickness skin tissues from infants, collected at the time of medically necessary surgery, and from “steady-state” adult tissues (buttocks) that had been protected from the effects of ultraviolet radiation. We hypothesized that epidermal barrier genes would be upregulated in adult skin, indicative of skin barrier homeostasis, relative to newborn infant skin where epidermal maturation is incomplete. Secondarily, we hypothesized that innate immune genes would demonstrate increased expression in newborn infant skin relative to adult skin that would reflect adaptive immune function.

## Materials and methods

### Subjects

Newborn infants were recruited from the Level IV Neonatal Intensive Care Unit (NICU) of Cincinnati Children’s Hospital Medical Center. Enrolled infants were expected to require surgery for their medical condition. They were a subset of infants participating in a larger study to examine skin barrier maturation. The Institutional Review Board of Cincinnati Children’s Hospital Medical Center approved the research. Parents granted written informed consent. Infants were excluded from participation if they were < 24 weeks GA, had cutaneous pathological conditions (e.g., ichthyosis, epidermolysis bullosa), were expected to be discharged before the surgical procedure and were medically unstable (i.e., could not undergo study procedures). The trial was registered in ClinicalTrials.gov Identifier: NCT01619228.

During the research design phase, the investigative team decided that it would be inappropriate to request parental participation for the collection of full-thickness tissue samples. The parents were focused on the care of their infants. Adult ultraviolet radiation protected samples (buttocks) from a separate clinical investigation were available for comparison to the infant samples from the present study. Previously, the molecular changes were reported for the female adult buttock samples versus facial skin samples (ultraviolet radiation exposed) over six decades of life [2]. Biopsy samples from adults aged 20–24 years and 60–64 years, representing ages of typical study parents and grandparents, were selected for comparison. Partners Human Research Committee Institutional Review Board approved the study and subjects provided written informed consent before any procedures were conducted. Subjects consented to the use of their tissue samples in other studies.

### Tissue collection

Infant full-thickness tissue collection sites were dictated by the surgical procedure, and included abdomen, chest, back, head and scrotum. The tissues were immediately placed in liquid nitrogen, snap frozen and transferred to the -80°C freezer. Adult 4-mm full-thickness biopsies collected from buttocks sites free of visual damage, e.g., rash, scars, dyspigmentation.

### Tissue processing and total RNA isolation

Frozen infant and adult tissue samples were placed in Trizol Reagent (Life Technologies, Waltham, MA), homogenized with a Polytron PT6100 homogenizer (VWR, Radnor, PA) and frozen overnight. The next day, samples were thawed, and insoluble materials were removed. The supernatant was mixed with chloroform, transferred to a Phase Lock Gel tube (5-Prime, Gaithersburg, MD) and centrifuged. Ethanol was added to the upper aqueous phase in a fresh tube, vortexed and transferred to an RNEasy mini column (Qiagen, Valencia, CA). The column was centrifuged and washed per manufacturer’s instruction. Total RNA (containing mRNA) was eluted from the column by centrifugation with preheated (65°C) nuclease-free water.

### Affymetrix GeneTitan mRNA target labeling, processing and analysis

Messenger RNA analysis was performed on purified RNA converted to biotin-labeled complementary RNA copies with the Affymetrix HT 3’ IVT Plus kit (Affymetrix, Santa Clara, CA) per protocol provided at a Beckman Biomek FXP Laboratory Automation Workstation (Beckman, Indianapolis, IN). Briefly, 250 ng of total RNA were reverse-transcribed into complementary DNA copies using oligo-dT primers and reverse transcriptase followed by second-strand synthesis using DNA polymerase I [2]. After purification, the cDNA library was used as a template to generate biotin-labeled cRNA copies using T7 RNA polymerase and biotinylated deoxyuridine triphosphate. Biotinylated cRNA was fragmented by limited alkaline hydrolysis and then hybridized overnight to Affymetrix GeneTitan U219 array plates using the Affymetrix GeneTitan instrument and protocol. After processing, chip images were converted to numeric data with the probe logarithmic intensity error algorithm as executed in the Affymetrix GeneChip Expression Console. All data were MIAME compliant.

### Validation of transcriptomics data

As the present study was a pathway identification analysis rather than a biomarker generation analysis, quantitative RT-PCR was not performed. In our experience, the size of the study, the overall study design, and the co-regulation of multiple members of the same pathway provide reasonable assurance that the data are reliable. This level of confidence has been established through multiple validation/correlation analyses over the past 20 years, both published [20–23] and unpublished. These correlation studies include comparisons with quantitative RT-PCR, RNASeq, Immunocytochemistry, and Luminex microsphere hybrid capture platform. A detailed discussion of the rationale is provided (S1 Document).

### Statistics, bioinformatics and data presentation

The data from all tissue samples were evaluated with rigorous quality control procedure to detect potential outliers from processing, instrumentation, or other reasons. This process included examination of the GeneChip level Affymetrix QC metrics (Affymetrix): Raw Q, Scaling Factor, Noise Average, and Background Average. Probe set level QC metrics, Prediction Interval Analysis, Principal Components Plots, and Pairs Plots, as well as Leave-One-Out analyses were also performed to ensure high quality data. A significance of adjusted P < 0.01 was used to identify genes that showed an increase or decrease of expression between groups. Infants and adults were compared and examined by differential expression analysis, identifying increased or decreased expression between infants relative to adults and vice-versa.

The 49,386 probes on the U219 chip were filtered to remove the lowest 30% of signals leaving 34,571 for analysis. Principal component analyses, MA-plots and Leave2Out prediction intervals were used to assess array quality. Samples were considered statistical outliers if they were visual outliers for the 1^st^ or 2^nd^ principal component of needle plots of Affymetrix QC plots, had a larger number of outliers for Leave2Out prediction intervals or had a large deviation from the all-samples means for a large number of probesets. Data were normalized using the PLIER (Probe Logarithmic Intensity ERror) algorithm to adjust for background noise. Log2 transformed data were used to compare infant and adult groups. A linear model was fit for each probe, testing for differences between age groups (infant, adult). To assess the need for controls, a principal component analysis (PCA) was performed and subjects were clustered based on anatomical site and sex. The decomposition method of PCA allows the user to cluster based on the similarity of all probes and showed no strong separation between either site or sex. Additionally, a differential expression analysis was performed. It showed only a minimal number of differentially expressed probes between site and sex. As a result, a simple model including age group was fit. Comparisons were tested using the Empirical Bayes method available in the limma R-package. Test statistics were moderated using the empirical bayes method and false discovery rates were controlled using the Benjamini-Hochberg correction. At significance P < 0.05, 2,491 probesets are expected to be significant by change only.

Hierarchical clustering was applied with the complete linkage method using the R hclust function. Significantly expressed genes were analyzed for enrichment of biologic themes (GO and KEGG pathway) through the use of the clusterProfiler package [24] String database [25], g:profiler [26], EnrichmentMap [27] and Revigo [28]. FDR adjusted p-values were used to select significant pathways. Heatmaps were generated on a standardized signal value by the R ComplexHeatmap packages [29]. For each gene across all samples, a z-score was calculated as (signal of the gene–mean signal from all samples)/standard deviation of the gene expression from all samples. Z-scores were displayed in the blue-white-red color gradient from -2 as the darkest blue, 0 as white, and 2 as the darkest red color. The transcriptomics data were deposited to the NCBI Gene Expression Omnibus (GEO) repository with the dataset accession number GSE181022.

## Results

### Subjects and tissues

Seventy-two infant patients were screened based on the expectation that they would have surgery. Of these, 27 were enrolled to provide 29 tissues; two infants had two surgeries. The anatomical sites were as follows: abdomen (n = 14), back (n = 6), chest (n = 7), head (n = 1) and scrotum (n = 1). Tissue sizes ranged from 3–4 mm x 1–2 mm. After quality control procedures, data from 27 of 29 tissues were carried forward for statistical analysis. Forty-three adult biopsies met the established quality criteria (Statistics, Bioinformation, Data Presentation). Infants were 36.1 ± 3.3 weeks gestational age (GA) at birth and 42.2 ± 11.4 weeks corrected age at tissue collection. Adults were 22.6 ± 1.5 and 62.3 ±1.5 years old, respectively (Table 1).

**Table 1:** Demographic characteristics of infant and adult subjects.

	Infants: weeks Mean ± SD (Median)	Adults 20-years-old: years, Mean ± SD (Median)	Adults 60-years-old: years, Mean ± SD (Median)
Number	27	25	18
Gestational Age (weeks)	36.1 ± 3.3	NA	NA
	(36.3)		
Age at Time of Collection (*Corrected Gestational Age at Collection for Infants)	42.2 ± 11.4 weeks	22.6 ± 1.5 years	62.3 ± 1.5 years
	(39.5)	(23)	(62.5)
Weeks from Birth at Tissue Collection	6.0 ± 10.6	NA	NA
	(1.7)		
Gender (M/F)	15/12	0/25	0/18
Race (Black/White)	4/23	0/25	0/18

*Corrected Gestational Age (GA) is calculated as the GA at birth (weeks) plus the time from birth (weeks). For example, an infant of 36 weeks GA who is 3 weeks old has a corrected GA of 39 weeks.

Most infant tissues were collected shortly after birth (median of 1.7 weeks) with a mean of 6.0 weeks of life. The mean value for age at collection in infants was higher due to inclusion of 4 subjects who were 14 weeks of age or older (14, 15, 28, 49). The time of surgery depended on the specific medical diagnosis and infant’s acuity.

### Gene expression and hierarchical cluster analysis of infants versus adults

**Fig 1:**
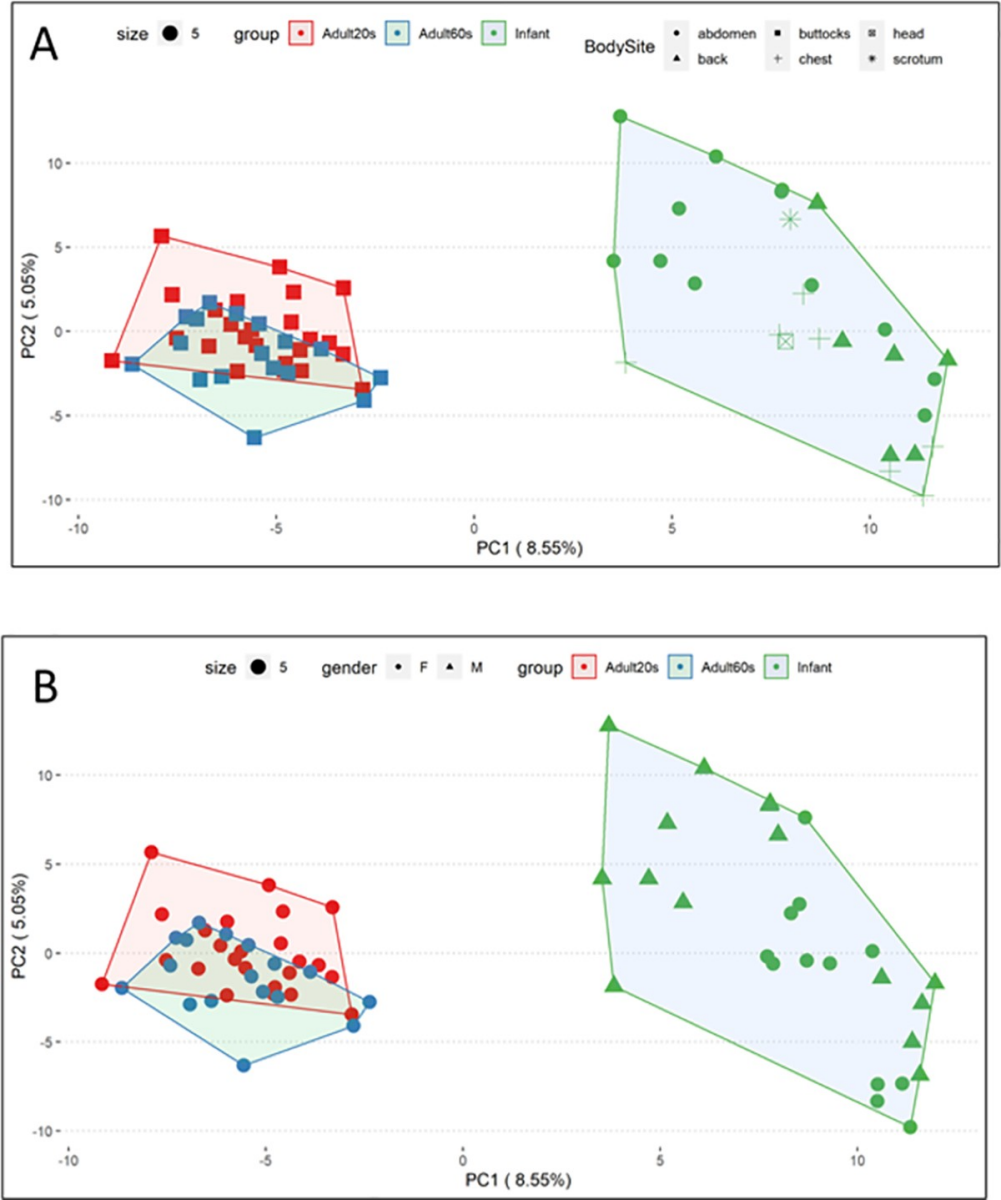
**A.** PCA plot showing adult groups, infants and infant anatomical sites. PCA plot shows Adults 20s (red squares), Adults 60s (blue squares), and Infants (green symbols). Within the infant cluster, anatomical sites are shown with different symbols (circle indicates abdomen, triangle indicates back, cross indicates chest). Clear separation in gene expression is observed for infant versus adult skin. The effect of gender is shown in Fig 1B, indicating strong separation between adults. **B.** PCA plot showing adult groups, infants and infants by sex. The PCA plot shows Adults 20s (red circles), Adults 60s (blue circles) and Infants (green symbols). Within the infant cluster, female sex is shown as circle and male sex as triangles. No strong separation in gene expression is observed in the infant cluster based on sex.

Statistical analysis established over 25,000 differentially regulated probesets, representing 10,647 distinct genes, in infant skin compared to adult skin, substantially more than expected by chance (See S1 File). For the total probeset (n = 49386), the number of differential probes (adjusted P ≤ 0.05) were (a) 25540 for all adults versus infants, (b) 20080 for adults 20 years old versus infants and (c) 146 for adults 20 years old versus adults 60 years old. The principal component analysis (PCA) method permits clustering based on similarity of all probes. Fig 1A shows the PCAs of the gene expression data for infants versus adults. Overlap in the adult population and clear separation in gene expression for infants versus adults can be seen. Analysis of the effect of anatomical site among the infants indicated no strong separation between sites (Fig 1A).

Within the infant cluster, no evidence is found conforming strong separation between females and males. The differential expression analysis showed only a minimal number of differentially expressed probes between site and sex. Consequently, a simple model including age group was fit. Given the similarities in gene expression within the non-UV exposed adult tissues, adult data were combined irrespective of age for all further analyses (Fig 1A and 1B).

Using a fold change (│FC│) ≥1.5, significant at adjusted P ≤0.05, 1,086 differentially expressed probes (DEGs, 654 unique genes) were identified in infant skin versus adult skin. Of these, 508 probes (329 unique genes) were increased and 578 probes (325 unique genes) were decreased.

**Fig 2:**
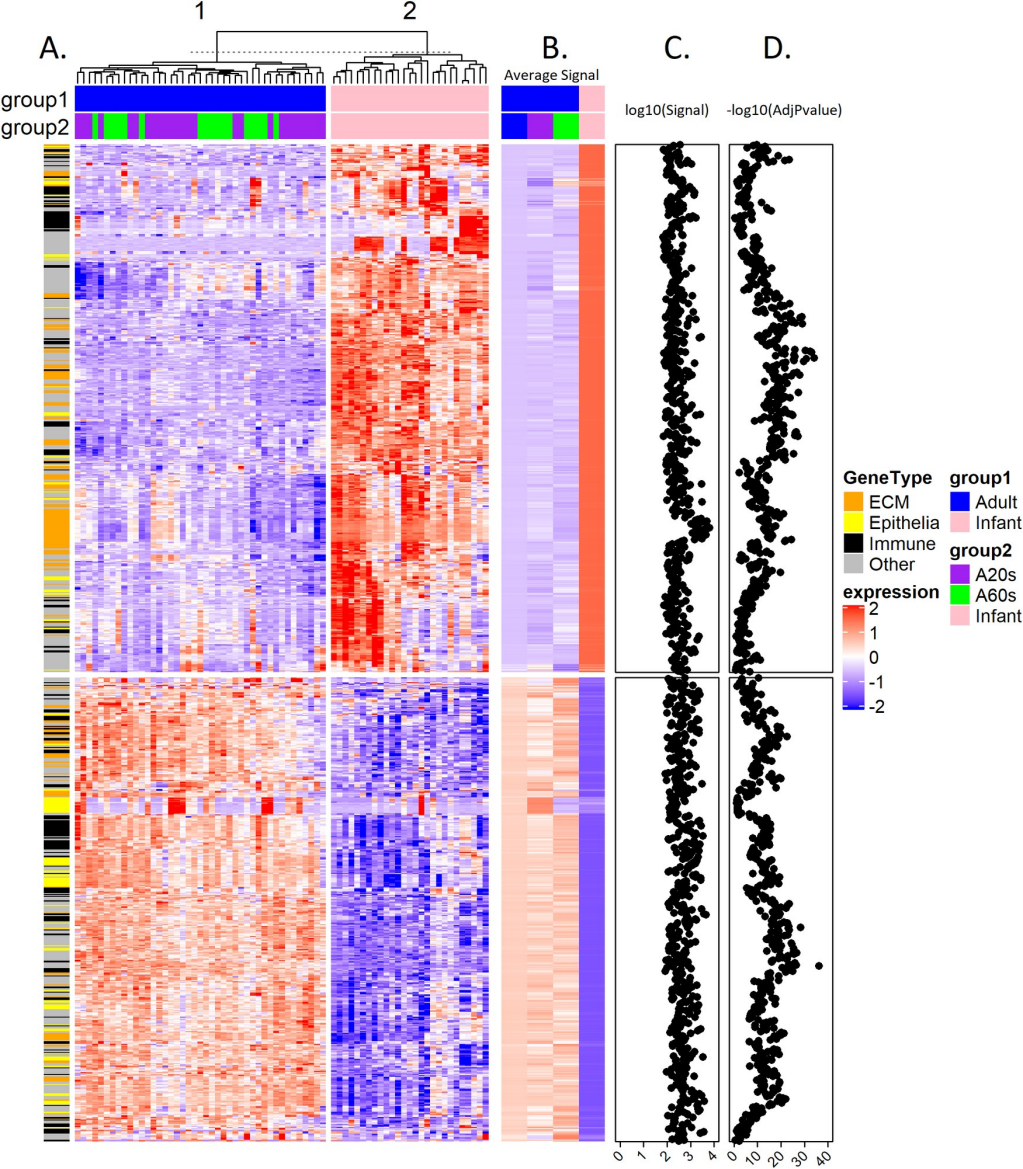
Hierarchical clustering analysis of differentially expressed genes in full-thickness skin tissue for infants and adults. A. Heatmap showing samples with normalized expression values (based on z-score) of all 1,086 differentially regulated genes with adjusted P value <0.05 and absolute fold change > = 1.5 for infant and adult (A20s: 20 years old, A60s: 60years old) Euclidean distances between each sample were calculated, and an unsupervised hclust algorithm was used for clustering analysis. Samples formed 2 clusters clearly separating infants (pink) and adults (blue, all adults; purple, 20 years; green, 60 years). Genes were grouped for similarity using hierarchical clustering analysis. Each column represents one sample and each row is a single gene. Extracellular matrix (ECM, orange), Immune-related (black) and Epithelial (yellow) genes are indicated at the left side annotation bar. B. Heatmap of group average normalized expression values are shown here. with significant differences between infant (pink) and adult (blue all adults; purple 20 years; green 60 years) groups. C. Log_10_ values of the all-sample average expression signals were plotted for each gene. D. Negative log_10_ of the adjusted P-value of the Limma testing for adult vs. infant comparison was plotted for each gene. Many of the negative log_10_(AdjPvalue) values are over 10, indicating large differences (high significance). The z-score is displayed in the blue-white-red color gradient from -2 as the darkest blue, 0 as white, and 2 as darkest red color.

The results of hierarchical clustering analysis of the normalized expression values (based on z-score) of the 1,086 probes (│FC│ ≥1.5 adjusted P value <0.05) for infant and adult skin samples are shown in Fig 2A–2D. Euclidean distances between each sample were calculated, and an unsupervised hclust algorithm was used for clustering analysis. Samples formed 2 clusters clearly separating infants and adults. Genes were grouped for similarity using hierarchical cluster analysis. Fig 2B shows the heatmap of the group’s average expression. Fig 2C shows the log_10_ value of the all-sample expression signals for each gene. Fig 2D is the negative log_10_ of the adjusted P value of the Limma testing for the infant and adult comparisons for each gene. Many of the negative log_10_(AdjPvalue) values are over 10, indicating large differences (high significance).

### Gene set enrichment analysis: Infant skin

Infant skin samples, when compared to adult skin, were enriched in genes associated with several biological processes including extracellular matrix (ECM) organization, collagen fibril organization, sulfur compound biosynthetic process and fatty acid metabolic process (Table 2). A total of 143 Gene Ontology (GO) themes were found for Biological Processes (BP) enriched in infants (P < 0.01). ECM organization and ECM structure organization had the lowest P values. Other significant processes included collagen, system development, regulation, response, e.g., to growth factor, bacterium, lipid or fatty acid, biosynthesis and metabolism (S1 Table). Of the 15 statistically significant GO themes for cellular components, the lowest P-values were ECM, collagen-containing ECM and endoplasmic reticulum lumen (S1 Table).

**Table 2:** Selected Gene Ontology (GO) biological themes with enriched gene expression in infant skin.

Term ID	Description	Significantly Changed Gene Count	P adjusted
GO:0030198	Extracellular matrix organization	44	2.37E-24
GO:0001568	Blood vessel development	38	9.61E-10
GO:0030199	Collagen fibril organization	12	7.97E-09
GO:0044272	Sulfur compound biosynthesis process	15	2.06E-05
GO:0006631	Fatty acid metabolic process	21	2.16E-05

Infant processes are listed in decreasing order of significance of over-representation of the regulated genes versus adults.

**Fig 3:**
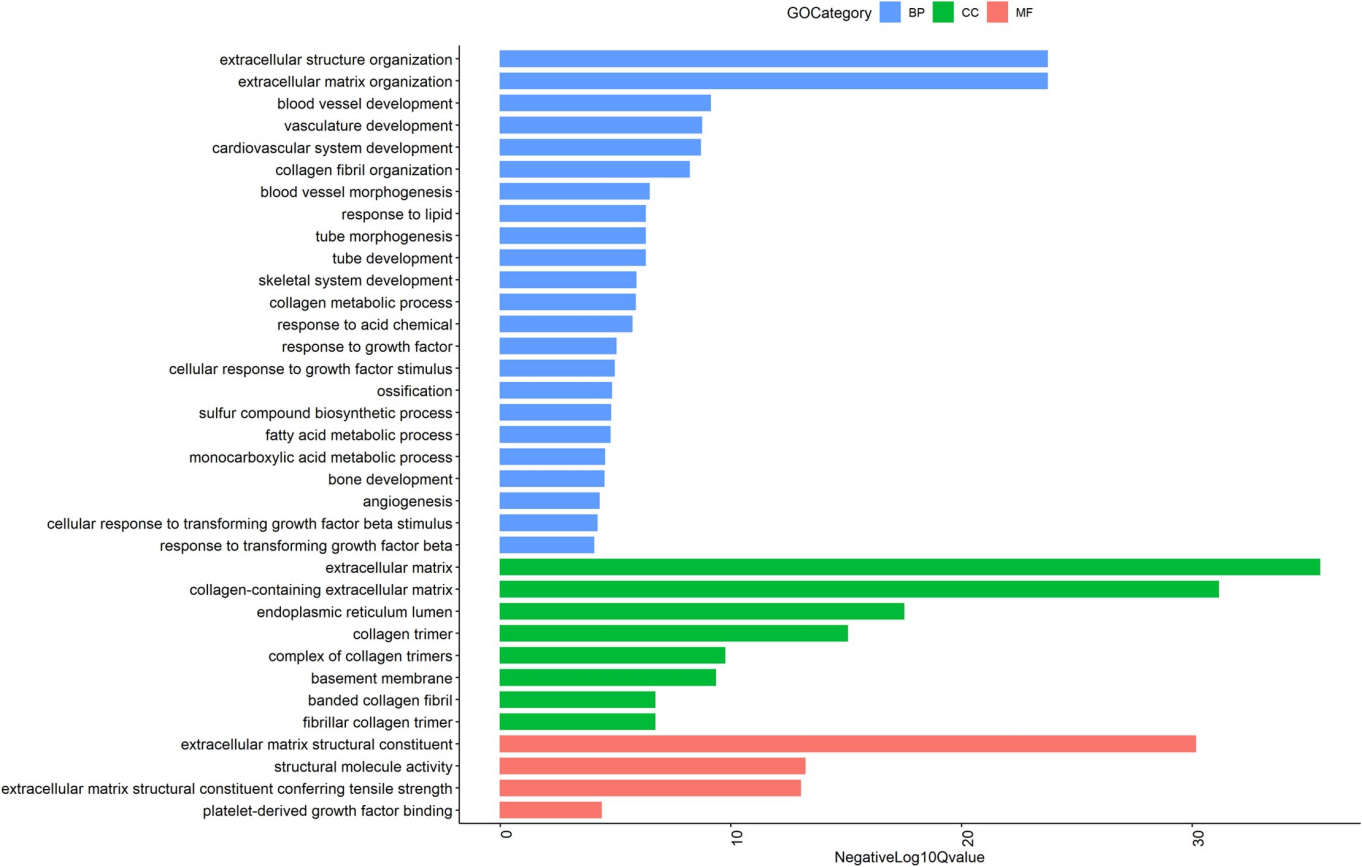
Results of the enrichment analysis against various databases including Gene Ontology for infant skin: Molecular Function (GO MF), Cellular component (GO CC), and Biological Process (GO BP) are shown for the terms with the lowest adjusted P-values in the infant samples. FDR adjusted P-values were used to select significant pathways. NegLog_10_Qvalue indicates -Log_10_FDR adjusted P-value.

Of the 35 increased GO themes for molecular functions, the most significant were ECM structural constituent, structural molecule activity, and ECM structural constituent conferring tensile strength. Five involved ECM and 18 were binding functions. Fig 3 shows Gene Ontology terms with the lowest adjusted P-values for infant skin. An enrichment map showing the genes for infant skin compared to adult skin is provided in S1 Fig. Significant biological themes from G:Profiler analysis with adjusted P-value <0.00001 are displayed in nodes. Edges were shown with similarity > = 0.5 between two nodes.

**Fig 4:**
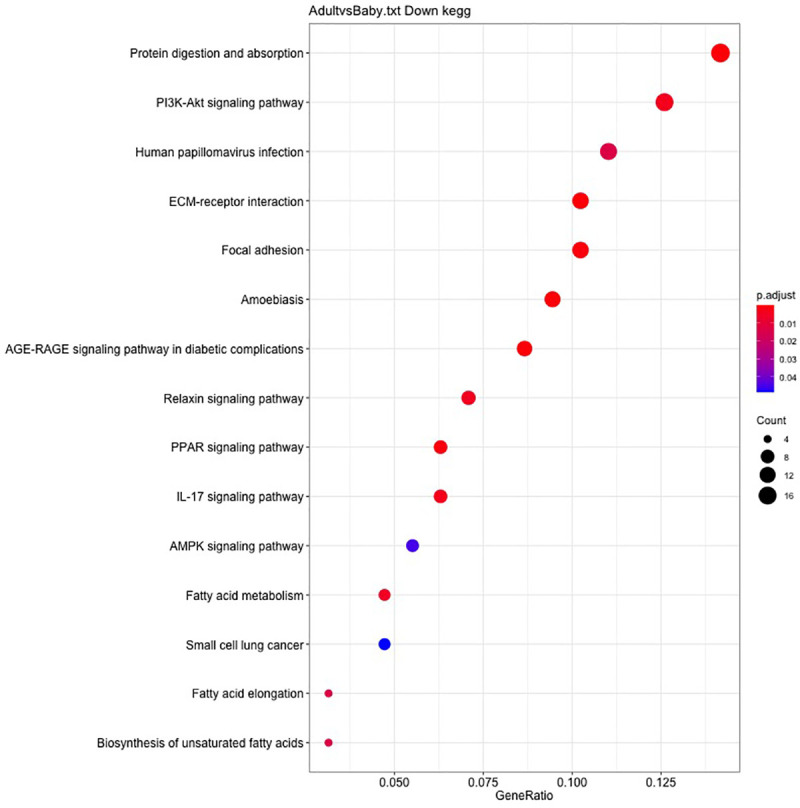
Results of the Kyoto Encyclopedia of Genes and Genomes (KEGG) pathway enrichment analysis (GeneRatio) are shown for the fifteen most significant pathways in infant skin. FDR adjusted p-values were used to select significant pathways. Dot size represents the number of genes in each pathway (Count); p.adjust (FDR adjusted P-value) and red < purple < blue.

Kyoto Encyclopedia of Genes and Genomes (KEGG) pathway analyses were conducted to examine signal transduction and metabolism. Fifteen pathways were significantly enriched; protein digestion and absorption and ECM-receptor interaction had the lowest adjusted P-values (Fig 4). Others included lipid metabolism, endocrine system/metabolism, infection, signaling and immunity. KEGG pathway maps for ECM-receptor interaction and fatty acid elongation are shown in S2 and S3 Figs.

### Gene set enrichment analysis: Adult skin

**Fig 5:**
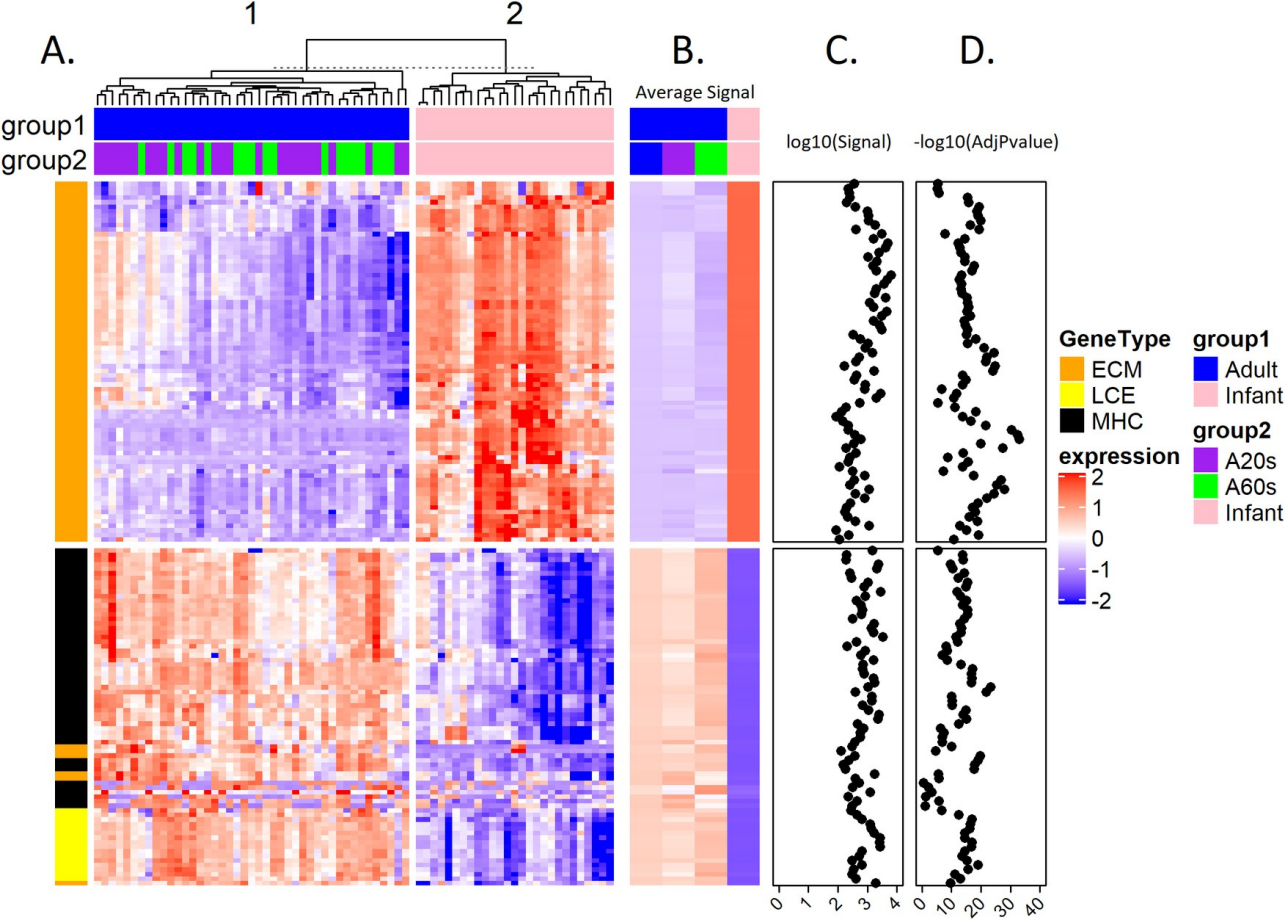
Hierarchical clustering analysis of differentially expressed genes for ECM, LCE and MHC in full-thickness skin tissue for infants and adults. A. Heatmap of samples showing normalized expression values (based on z-score) of selected differentially regulated genes with adjusted P value < 0.05 and absolute fold change > = 1.5 associated with ECM, LCE, and MHC for infant and adult (A20s: 20 years old, A60s: 60 years old). Euclidean distances between each sample were calculated and an unsupervised hclust algorithm was used for clustering analysis. Samples formed 2 clusters clearly separating infants (pink) and adults (blue, all adults; purple, 20 years; green, 60 years). Genes were grouped for similarity using hierarchical cluster analysis. Each column represents one sample and each row is a single gene. Infant samples have higher ECM gene expression (orange) and lower MHC (black) and LCE (yellow) expression than adult samples. B. Heatmap of group average normalized expression values is shown here with significant differences between infant (pink) and adult (blue all adults, purple 20 years; green 60 years) groups. C. Log_10_ values of the all-sample average expression signal were plotted for each gene. D. Negative log_10_ of the adjusted P value of the Limma testing for adult vs. infant comparison were plotted for each gene in the ECM, LCE and MHC classes. Many of the negative log_10_(AdjPvalue) values are over 10, indicating large difference (high significance). The z-score is displayed in the blue-white-red color gradient from -2 as the darkest blue, 0 as white, and 2 as darkest red color.

Genes associated with epidermal development, keratinocyte differentiation, immune function (antigen processing and presentation of exogenous antigen) and hair cycle (Table 3) were expressed in adult skin compared to infant skin. A total of 101 enriched GO themes for biological processes (adjusted P-value < 0.05) were observed. The 8 with the lowest adjusted P values involved skin and epidermal development (keratinocyte differentiation, keratinization, cornification) as well as intermediate filament cytoskeleton organization and hair cycle. More than half (n = 58) of the GO biological processes concerned immune function, including 17 antigen processing and presentation processes (S2 Table). The 64 increased GO themes for cellular components (adjusted P-value < 0.05) in adult skin involved the epidermis, including cornified envelope, keratin filament and desmosome, and the immune system (S2 Table). Fig 5A–5D compare the expression in infants and adults for genes associated with ECM, late cornified envelope (LCE) and major histocompatibility complex (MHC). Euclidean distances between each sample were calculated and an unsupervised hclust algorithm was used for clustering analysis. Samples formed 2 clusters clearly separating infants and adults. Genes were grouped for similarity using hierarchical clustering analysis. Fig 5A and 5B are heatmaps of relative gene expression. Fig 5C shows the log_10_ values of the all-sample expression signal for each gene. Fig 5D shows the negative log_10_ of the adjusted P value of the Limma testing for the infant and adult comparison for each gene in ECM, LCE and MHC. Many of the negative log_10_(AdjPvalue)values are over 10, indicating large differences (high significance).

**Table 3:** Selected Gene Ontology biological themes with enriched gene expression in adult skin.

Term ID	Description	Significantly Changed Gene Count	P adjusted
GO:0008544	Epidermis development	51	3.48E-26
GO:0030216	Keratinocyte differentiation	42	1.65E-25
GO:0019884	Antigen processing and presentation of exogenous antigen	19	1.30E-08
GO:0042633	Hair cycle	10	7.10E-04
GO:0030141	Secretory granule	28	4.89E-04
GO:0004866	Endopeptidase inhibitor activity	11	2.88E-03
GO:0008236	Serine-type peptidase activity	10	8.18E-03

Adult processes are listed in decreasing order of significance of over-representation of the regulated genes versus infants.

**Fig 6:**
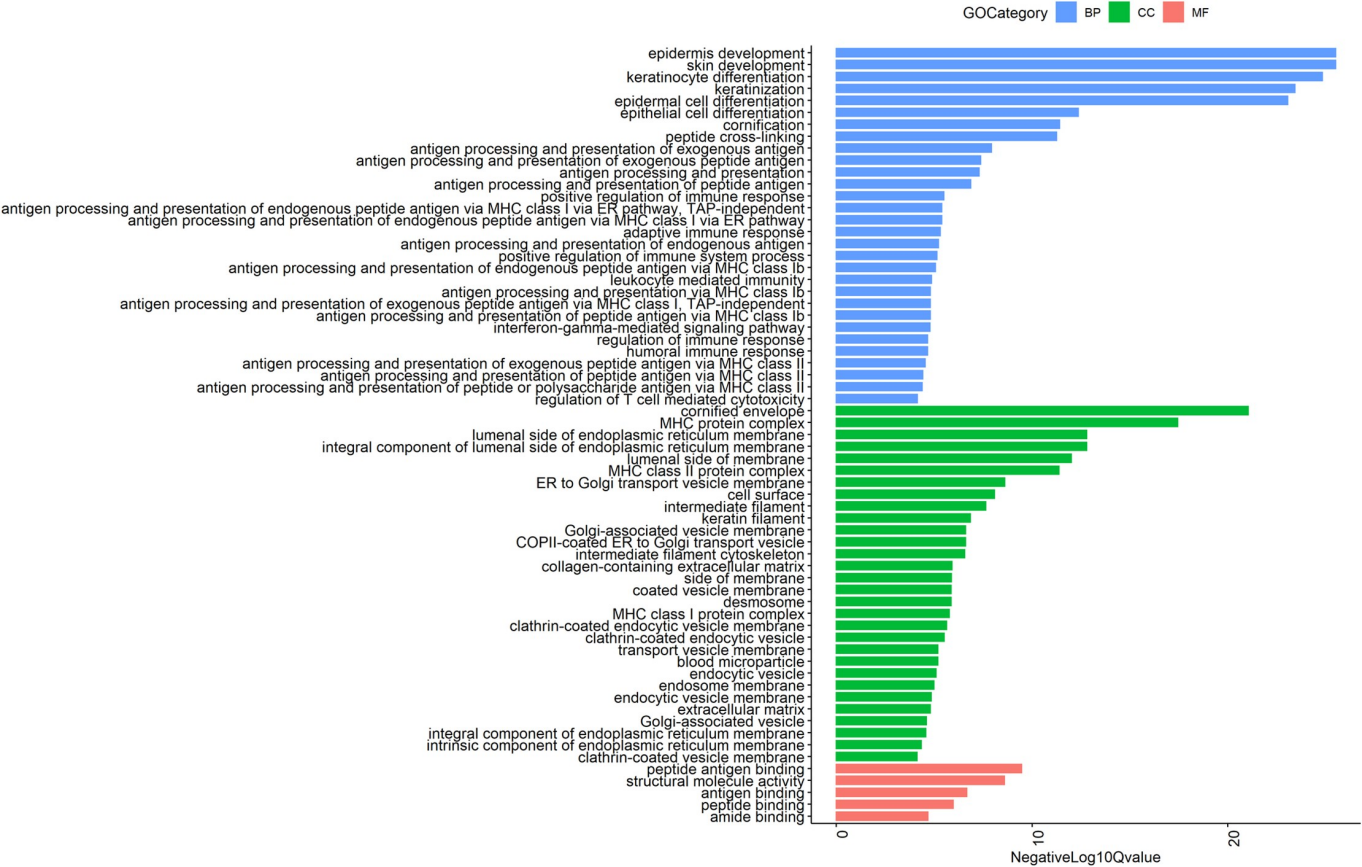
The results of the enrichment analysis against various databases including Gene Ontology for adult skin: Molecular Function (GO MF), Cellular component (GO CC), and Biological Process (GO BP) are shown for adult skin with the top most significantly increased terms. FDR adjusted P-values were used to select significant pathways. NegLog_10_Qvalue indicates -Log_10_FDR adjusted P-value.

Peptide antigen binding was the most significant of 22 enriched GO themes for molecular functions in adult skin when compared to infant skin. Ten were binding functions and 10 involved enzymatic activity or inhibition. Fig 6 shows the enriched Gene Ontology terms with the lowest adjusted P values in adult skin. An enrichment map showing the genes for adult skin compared to infant skin is shown in S4 Fig. GO themes for biological processes from G:Profiler analysis with adjusted P value <0.00001 are displayed in nodes Edges are shown with similarity > = 0.5 between two nodes.

**Fig 7:**
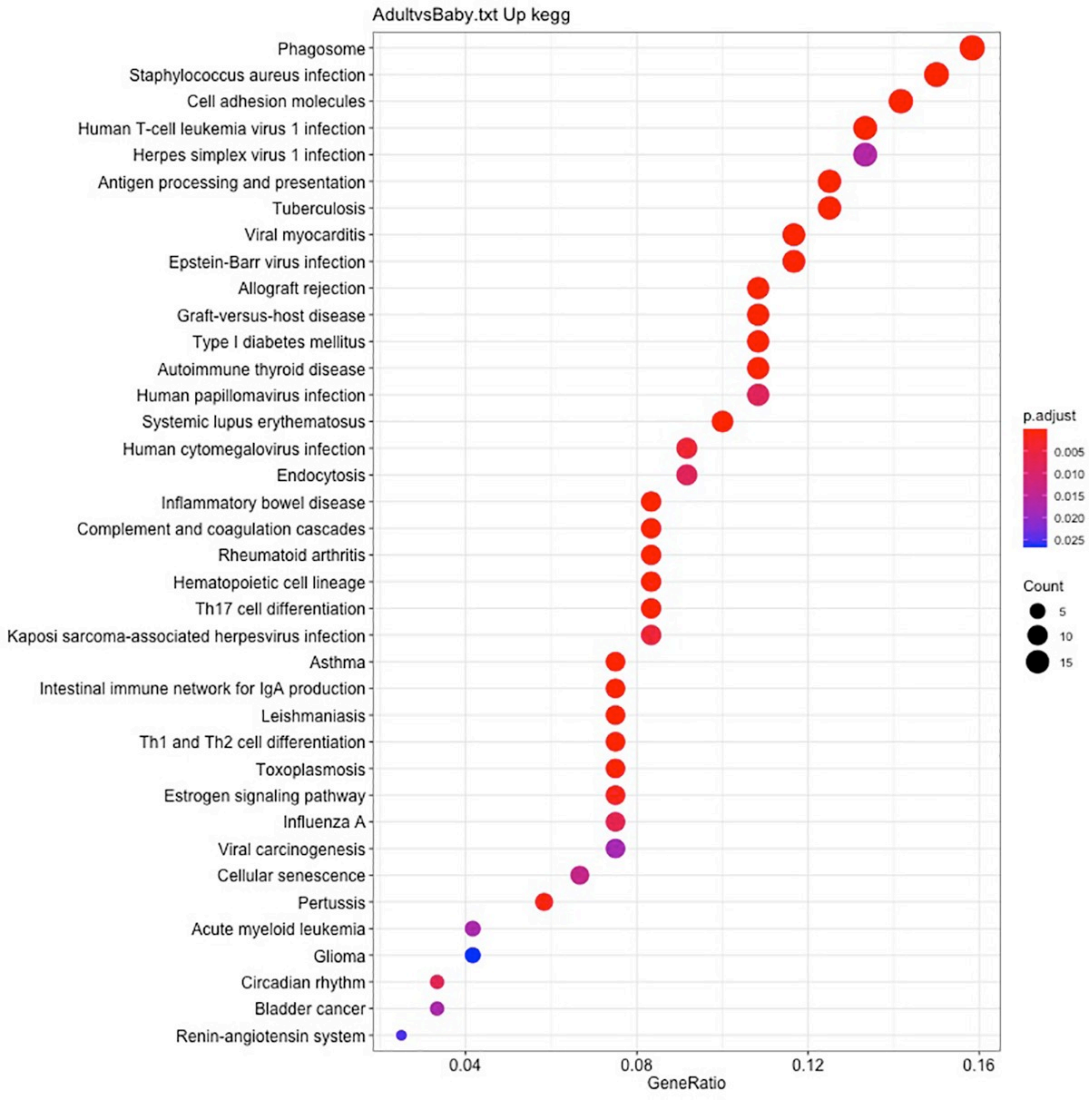
Results of the Kyoto Encyclopedia of Genes and Genomes (KEGG) pathway enrichment analysis (GeneRatio) are shown for the thirty-eight most significant pathways in adult skin. FDR adjusted P-values were used to select significant pathways. Dot size represents the number of genes in each pathway (Count); p.adjust (FDR adjusted P value) and red < purple < blue.

Thirty-eight KEGG pathways were enriched for adult skin and the lowest adjusted P-values were for Staphylococcus aureus infection and allograft rejection (Fig 7). Others were immune system or immune disease, infectious disease, cancer, transport and catabolism, and endocrine or endocrine disease. S5 and S6 Figs show pathway maps for *Staphylococcus aureus* infection and antigen processing and presentation.

### Individual gene classes: Infant skin versus adult skin

To further explore the differences between infant and adult skin samples, we also examined the differentially regulated genes (same statistical criteria, adjusted P-value ≤ 0.05) by considering gene families. The results are shown in Table 4.

**Table 4:** Comparative overexpression for selected gene families for infant skin versus adult skin.

Gene Class	Elevated Expression in Infant Skin	Elevated Expression in Adult Skin
Collagen	COL4A1, COL4A2, COL4A5, COL4A6, COL5A1, COL5A1, COL1A1, COL1A2, COL3A1, COL5A3, COL15A1, COL14A1, COL6A1, COL6A2, COL6A3, COL11A1, CTHRC1, CCBE1, COLGALT1	COL17A1, COL8A2
Elastin	ELN, EMILIN1	none
Matrix metalloproteins	MMP 7, 14, 16, 19, 27	MMP 2, 9, 28
Fibronectin	FNDC3B, FNDC3A, GNDC1, FLRT2, FLRT3	none
Integrin	ITGB1, ITGA6, ITGAV, ITGA7, ITGA9, ITGB8, ITGB3BP	ITGB2, ITGB4, ITGB5, ITGAM, ITGBL1, ILK, ITFG2, ITGB1BP1
Laminin	LAMB1, LAMC1, IBR, IMNB2, LAMA4	LAMB3, LAMC2, LMNA, LAMC3, LAMA5
Nidogen	NID1, NID2	none
Proteoglycan	PRG4	HSPQ2, PAPLN
Major histocompatibility complex	HLA-A, HLA-B, HLA-C, HLA-DMA, HLA-DMB, HLA-DOA, HLA-DOB, HLA-DPA1, HLA-DPB1, HLA-DQA1, HLA-DQB1, HLA-DRA, HLA-DRB1, HLA-DRB4, HLA-E, HLA-F, MR1	none
Heat shock protein	HSPD1, HSP90B1, HSPA5, HSPH1, HSP90AA1, HSPA4, HSPA8, HSPA9, HSPA13, HSPE1, HSP90AA4P, HSBP1, HSPA12A, HSP90AB1	HSPA2, HSPB8, HSPB2, HSPA14, HSPB1, HSPA6, HSF4, HSPB7, HSPB11, HSBP1I1
Chemokine	CCL2, CCL21, CXCL2	CCL12, CCL18, CCL19, CCL27, CCL3P1, CKLF, CX3CR1, CXCL12, CXCL14, CXCL16, CXCL5
Interleukin	IL6ST, IL33, IL11RA, IL1R1, IL1R2, ILF3, IL17RB, IL17RD	IL1RAP, IL1RN, IL2RB, IL6R, IL10RB, IL13EA1, IL16 IL18, IRAK1, IL17RA, IL36RN, IL37, ILAK4, IL23A, IL20RA, IL20RB, IL22RA1, IL17RC, IL17RE, IL34
Proteasome	PSMD12, PSMA4, PAMC6, PSME4, PSMB5, PSMA7, PSMA1, PSME2, PSMB2, PSMD1	PSMB1, PSMB3, PSMB4, PSMB6, PSMB7, PSMB8, PSMB9, PSMB10, PSMC1, PSMC2, PSMC3, PSMC5, PSMD2, PSMD3, PSMD4, PSMD5, PSMD7, PSMD8, PSMD9, PSMD11, PSMD12, PSMD13, PSME1, PSMF1, PSMD6, PSME3, PSMG2, PAAF1, PSMG3, PSMG4
Ubiquitin	UBB, UBE2D1, UBE2H, UBE2I3, UBE2V2, UQCRC2, UQCRH, USP1, USP6, USP10, UBE4A, USP34, USP15, UBA2, USPL1, USP16, UBE2C, USP3, UBE2S, UBE2T, UQCR10, UBQLN2, UBR5, UFM1, UBA6, UBE2W, UBFD1, USP46, UBA5, USP48, UPCC2, USP38, USP32, ZNF587, UBTD2, UBE2Q2, UHRF2, UBR3, UBE2F, UBR1,	UBL3, UBA52, UBC, UBA1, UBA7, UBE2D2, UBE2E1, UBE2E2, UBE2G1, UBE2I, UBE3A, UFD1I, USP4, UQCRC1, USP7, USP5, USP9X, UBL4A, UBE2M, USP14, USP8, UBE2I6, UBE4A, UBE3C, UBAP2I, USP15, USP3, UBE4B, UBE2E3, USP39, USP19, UBR4, USP21, UBQLN1, UFC1, USP53, UBE2R2, UBR7, UBE2Q1, UBQLN4, USP31, USP36, UBL5, USP30, UBL7, UBE2J2, USP54, USP12, UPCC3
Serine protease inhibitors	SERPINE2, SERPINF1, SERPINB3, SERPINH1, SERPINB9	SERPINA3, SERPING1, SERPINB1, SERPINB5, SERPINB7, SERPINB8, SERPINA12

As expected from the GO theme results, none of the MHC genes had increased expression in infants. Collagen, fibronectin, nidogen, and elastin genes were generally increased in infants relative to adults, while increases in the integrin and laminin genes occurred for both groups.

Chemokine, interleukin and proteasome family genes were relatively increased in adults, while heat shock proteins, ubiquitin and serine protease inhibitors were comparable between infants and adults.

### Specific genes of interest in infant skin

Genes involved with “adhesion”, specifically desmogleins DSG2 and DSG3, were highly expressed in infant skin while DSG1 expression was higher in adult skin. Desmocollin DSC2 was higher in infant skin, while DSC 1 and DSC3 were higher in adult skin. Tight junction genes TJP1 and TJP2 were higher in infant skin. The ADAMST genes, i.e., regulators of proteases involved with fibrillin microfibril formation and function [30], ADAMST1, ADAMST4, ADAMST9 and ADAM9 showed increased expression in infant skin compared to adult skin.

Gene expression of PI3, S100A8, S100A9, S100A7, SERPINB3 and SERPINB4 are of particular interest based on our previous comparison of infant and adult SC samples using proteomic analysis [19]. PI3 regulates peptidase and endopeptidase activity. S100A8 regulates of endopeptidase and peptidase activity, cell death, inflammatory response, toxic substance response, lipid metabolism, bacterial response, wounding/wound healing and chemokine production. S100A9 is implicated in peptidase and endopeptidase activity, cell differentiation, cell death, inflammatory response, toxic substance response, bacterial response and chemokine production. SERPINB3 regulates peptidase and endopeptidase activity, cell differentiation, cell migration, cellular component movement, cell motility, and locomotion. In the present study, these four genes exhibited relatively higher expression in full-thickness infant skin samples. S100A7 and SERPINB4 were not differentially expressed in tissue.

## Discussion

We characterized the physiological, structural and functional features of newborn infant skin and compared them to protected adult sin (no exposure to ultraviolet radiation). Analysis of the 1,086 probes differentially expressed for infant skin versus adult skin revealed abundant differences across biological processes. Adult skin, when compared to infant skin, had increased gene expression for epidermal homeostasis, antigen processing/presentation of immune function, hair cycle and others (Figs 2 and 3; Tables 2 and 3). Infant skin, when compared to adult skin, had increased gene expression for several processes, including extracellular matrix, development, and fatty acid metabolism (Figs 2 and 3; Tables 2 and 3). To the best of our knowledge, this is one of a few reports to demonstrate considerable differences between full-thickness infant body skin within a few weeks of birth compared to UV-protected adult skin based on transcriptomic analysis. Holistically, the comparison of infant and mature, steady-state adult skin identified, three major differential biological processes: epidermal development, extracellular matrix and immunity. These findings can serve as guidelines toward preserving unperturbed young skin, understanding the trajectory of skin disease, such as atopic dermatitis, and mitigating the effects of environmental stressors.

Adult skin gene expression differed markedly from that of infant skin, particularly for processes regulating epidermal barrier formation and homeostasis and antigen processing and presentation (adaptive immune system). Relative increase in the expression of genes in these pathways in adult skin suggests that the infant epidermal barrier is under development, while adult skin is in homeostasis, i.e., undergoing constant renewal. In the presence of exposure to microbiota and dramatically different environmental conditions at birth, the infant relies on components of the innate immune system, including extracellular matrix, that prompt development of adaptive immunity.

### Epidermal maturation

Relative to infants, epidermal development was increased in adult skin as evidenced by the increased expression of genes in this process. This finding of ongoing epidermal barrier formation is consistent with continual renewal and replacement of the epidermis and SC. For infants relative to adults, gene expression for epidermal development was dysregulated, with the following GO biological processes having the lowest adjusted P values: skin development, epidermis development, keratinocyte differentiation, keratinization, epidermal cell differentiation, epithelial cell differentiation, cornification and peptide cross-linking (Figs 2, 5 and 6). For infants, gene expression was decreased for late cornified envelope genes LCE1A, LCE1C, LCE1D, LCE1E, LCE1F, LCE2A, LCE2B, LCE2C, LCE2D, LCE5A and LCE6A. Also reduced in infants were keratin genes, i.e., fibrous structural proteins associated with SC integrity: KRT2, KRT25, KRT27, KRT31, KRT74, KRT80, KRT85 and KRT86. Gene expression for KRTAP genes (KRTAP1-5, KRTAP3-1, KRTAP3-2, KRTAP4-3 and KRTAP4-9), associated with hair diversification and adaptation [31], was increased in adults [32, 33]. The differences in epidermal barrier gene expression are consistent with previous reports wherein measures of skin function, such as hydration, skin surface pH, TEWL, and visual dryness/scaling, have allowed drawing the conclusion that infant epidermal barrier maturation/adaptation continues for weeks to months following birth [32, 33]. They may also explain the relatively reduced SC cohesion and lower antimicrobial peptide levels soon after birth [19].

The lipid elongation pathway had increased gene expression in infant skin. ELOVL3 is involved with neutral lipid generation and maintenance of proper lipid ratios in the SC extracellular membrane [34]. Deficiency in ELOVL3 gene products results in barrier compromise, such as increased TEWL. ELOVL3, 5, and 6 were implicated in fatty acid elongation of saturated and branched chain fatty acids (in skin and vernix) [35]. Atopic skin was deficient in ELOVL3 and ELOVL6 proteins, suggesting that deficiency results in an abnormal lipid composition, i.e., lack of the longer chain fatty acids, leading to a defective barrier [36].

### Extracellular Matrix (ECM)

We asked why ECM genes were overexpressed in infant skin (Figs 2, 5 and 6) relative to adult skin. ECM genes regulate multiple processes including cell proliferation, differentiation, adhesion, migration, apoptosis [37], and barrier restoration [38]. The ECM is continually modified and renewed at high rates during development, wound repair, and infection [38]. ECM connects the epidermis and dermis via the basement membrane for structural integrity. In the developing fetus, periderm covers the epidermal basal layer and forms tight junctions during development to provide a barrier. When periderm regresses, fetal suprabasal cells adhere to other cells to create structure/barrier [38]. Consequently, loss of periderm may upregulate ECM genes to facilitate barrier development after birth.

Specific genes from the GO biological processes for extracellular matrix organization and extracellular structure organization (lowest adjusted P values) were considered. For example, ADAM9 gene expression was increased in infant tissues. It interacts with integrin-β1 on keratinocytes via adhesion to produce MMP9 and to regulate cell migration. After wounding in an animal model, ADAM9 regulated keratinocyte migration during healing by restraining collagen VII shedding [39]. During fetal development in a rat model, ADAMTS1 levels decreased, except for epidermis, teeth, and bone where it increased [40]. ADAMST1 was expressed, stored, and secreted in large amounts in keratinizing tissue (e.g., skin) and levels of ADAMST1 gene expression products were higher in animals at birth compared to adults [40], consistent with our findings. These mechanisms may be operational in newborn infants during the weeks after birth. Fibrillin gene expression (FBN1, FBN2) was increased in the infant skin. Fibrillin protein assembles into microfibrils and is reported to produce long-range tissue extensibility [41].

Fetal and adult skin differences have been reported regarding the robust wound repair rate and scarless healing in the fetus. Fetal and adult skin differed in ECM composition, gene expression, cytokine response, and inflammation [42]. TGFβ3 in fetal wounds is thought to block terminal cell differentiation and thereby halt inflammation [43, 44]. TGFβ3 expression was increased in our infant versus adult tissues. The ECM components fibronectin and chondroitin sulfate were increased in fetal versus adult skin and implicated in scarless wound healing [45]. We observed increased gene expression in FNDC3B, FNDC3A. GNDC1 FLRT2, FLRT3 from the fibronectin family, and ELN in infant skin versus adult skin.

### Immunity

The human immune system is immature at birth, but develops throughout childhood, adolescence and adulthood before diminishing with advancing age [46]. During gestation, the mother-child immune system is complex and uniquely adapted for the fetus has in order to withstand maternal alloantigens [47]. Infant survival depends on innate immune mechanisms to manage the transition.

The ECM has been implicated in immune response. When injury or infection occurs, immune cells produce enzymes, including MMPs, ADAMs, and ADAMTSs, that cause immune cells to migrate to the damage site and promote inflammation [48]. The ECM aids in immune response and immune cells assist in ECM repair. ECM proteins, including collagen, laminin, and fibronectin, bind to microorganisms that can degrade the ECM [49]. ECM processes, e.g., synthesis, assembly, remodeling and degradation, are controlled by immune cells and are actively involved in response to infection [50].

Xu, et. al. evaluated two adult groups, one with young-appearing skin and the other with appearance consistent with chronological age. They found differences in PDLA, HAS2-HA1 and immunological gene sets involving vaccine response, thymocyte development and FOXP3 which regulates development and function of regulatory T cells [51]. The immune genes were less expressed in youthful skin and more expressed in non-youthful skin, consistent with our findings for infants (younger individuals) versus adults.

A relevant comparison of both genomic profile and response to stress in neonatal versus adult animals was elucidated by Ubags, et. al. They examined skin and lung response to allergen exposure in neonatal and adult mice [52]. Adult animal lungs displayed T_H_2/T_H_17 inflammation and neonatal lungs showed T_H_2 inflammation. Unlike the adults, neonatal animals did not respond with skin inflammation. However, genes related to antigen-processing and presentation, were less expressed in neonatal versus adult animals, paralleling our finding for infant versus adult skin gene expression. A study of fetal and adult heart tissue reported an increased expression of genes in the antigen receptor-mediated signaling pathway in adults versus fetal samples [53], consistent with our findings of increased expression of antigen processing and presentation genes in adult skin.

Heat shock protein (HSP) gene expression was increased in infant skin versus adult skin (Table 4). Heat shock proteins form following stress, including temperature, pH, oxygen deprivation, and nutritional compromise. They serve as chaperones to facilitate protein folding/confirmation and minimize aggregation and denaturation [54]. HSPs can prompt the immune system to secrete cytokines or chemokines, trigger antigen presentation in dendritic cells [55] and prompt IL6, IL8 and TNFα production by monocytes [56]. HSPs activate adaptive immunity by providing peptides for major histocompatibility complex (MHC) loading and antigen specific responses [54]. HSPB1 gene expression was increased in the infants and is involved in late-stage keratinization. HSPA1 participates in cytoprotection with HSPA2 in early keratinocyte differentiation and HSPC in re-epithelialization [57]. Thus, HSPs may play a role in innate defense and drive epidermal barrier development prior to maturation of the adaptive immune system.

SERPINB3 gene expression was elevated in infant tissues, consistent with the previous report of the gene product being higher in the outer SC of infants versus adults [19]. Higher levels of SERPINB3 regulate cysteine proteases, and, later, transglutaminases and epidermal homeostasis [58]. They may also be involved in moderating an immune response [58].

### Extending the understanding of pediatric skin to infant skin

Transcriptomics analyses of pediatric skin samples have been published. Brunner et al. evaluated transcriptomic differences between normal and atopic dermatitis skin from children (n = 18, 1.2 ± 0.8 years) and compared those skin samples to normal and atopic dermatitis skin from adults (lesional and non-lesional sites) to determine the mechanisms underlying pediatric and adult AD [59]. Some overlap in gene expression was found for pediatric versus adult controls and further separation between pediatric and adult subjects was noted for lesional and non-lesional sites. For the controls (no AD), PI3 was increased in pediatric subjects, as were IL-6, CXCL8 and CCL20, while IL-33 was reduced, findings consistent with our observations (Table 4). In contrast, although we found ELOVL5 to be increased, it was a decreased in their pediatric patients versus adults.

Aging effects were investigated using foreskin tissue from boys aged 3–4 years (n = 5) and men aged 68–72 (n = 5) [60]. These investigators found 105 genes with expression at 1.7-fold or higher with 62 increased and 43 decreased. Genes PI3, S100A7, S100A8, S100A9, KRT6A, KRT16, STAT3 were increased in adults, in contrast to our study where they were increased in infants. Genes ST14, TGFα, MARK2, APOD, CTSD, PSMB9 and CTSL were decreased in the pediatric subjects and similarly decreased in our infants. These differences may reflect the variations in sampling sites and/or sample sizes.

Neonatal skin may be a relevant starting point for understanding what genomic changes precede pathologies such as atopic dermatitis (AD). An endotype for severe atopic dermatitis in children was characterized by reduced filaggrin (FLG) in non-lesional skin, suggesting that AD may begin with barrier dysfunction rather than the appearance of eczema (lesions) [61]. Pediatric subjects do not always manifest the characteristic atopic progression, emphasizing the need to establish the early physiological changes preceding atopic disease. Our infant results may assist in understanding and preventing the development of AD.

Some specific features were noteworthy, as they emphasize the utility of the findings and the limitations. We hypothesized that epidermal barrier genes would be upregulated in adult skin and that innate immune genes would demonstrate increased expression in newborn. The findings support them, yet the present study is largely descriptive, owing, in part to the large number of differentially expressed genes. Several sources of variability can be found within our infant sample, specifically anatomical location, sex and time from birth until tissue collection, that we were unable to control, given the medical status of the infants and the understandable concerns by parents about participation. Difficulties in collecting full-thickness skin from neonates within days of birth will likely persist. We examined the effects of anatomical site and infant gender during statistical model building, tested them to see if inclusion was necessary and determined that it was not. Model selection was done to save degrees of freedom whenever possible. The statistical analysis of sex effects among the infants indicated no clear separation among females and males. We were unable to collect buttock samples from male subjects. Therefore, the inability to incorporate the effects of sex for the adult versus infant comparison is a limitation of this study. Clearly, further studies to increase the sample size and, thereby, address the variables are warranted. We searched PubMed to examine previously reported information on gene expression of the multiple genes that were increased in newborn infant skin. Searches on the gene symbol and infant skin identified a limited number of papers, given the number of genes. They often reported on skin pathologies, such as atopic dermatitis [62–64]. A recent paper by Li, et al, describes a data mining method to identify key factors and genes in epidermal barrier development from the large number of publicly available databases [65]. They indicated that a considerable number of the genes already identified to control epidermal development have not been confirmed by experiments, such as loss- or gain-of-function studies. Therefore, additional experiments are needed to understand the impact of infant factors on specific gene sets or pathways on infant skin at birth.

Taken together, our data provide new and important information on the transcriptional changes occurring during the ontogeny of skin during the first few weeks of life. The skin is highly reactive and adaptive, but the types of environmental exposure immediately following birth have the potential to alter the “intended” programmed trajectory resulting in aberrant skin or diseased states. Therefore, it is critical to understand how the skin evolves from birth to old age in order to preserve newborn skin features or to rejuvenate adult skin.

## Supporting information

S1 Fig Enrichment map for the enriched gene sets in newborn infant skin compared to buttocks skin from adults of 20–64 years of age protected from ultraviolet radiation exposure. Significant biological themes from G:Profiler analysis with adjusted p value <0.00001 were displayed in nodes. Edges were shown with similarity > = 0.5 between two nodes.
(TIF)

S2 Fig KEGG pathway map for ECM-receptor interaction that was increased in infants. Blue color indicates significantly lower gene expression in adult skin versus infant skin. Pink color indicates significantly higher gene expression in adult skin versus infant samples. If multiple probes matched to the same gene, the one with smallest p value was selected to color the box.
(TIF)

S3 Fig KEGG pathway map for fatty acid elongation that was increased in infants. Blue color indicates significantly lower gene expression in adult skin versus infant skin. Pink color indicates significantly higher gene expression in adult skin versus infant samples. If multiple probes matched to same gene, the one with smallest P value was selected to color the box.
(TIF)

S4 Fig Enrichment map for the enriched gene sets in buttock skin that was protected from ultraviolet radiation exposure in adults of 20–64 years of age compared to newborn infant skin. Significant GO themes of biological processes from G:Profiler analysis with adjusted P value <0.00001 were displayed in nodes. Edges were shown with similarity > = 0.5 between two nodes.
(TIF)

S5 Fig KEGG pathway map for Staphylococcus aureus infection that was increased in adults. Blue color indicates significantly lower gene expression in adult skin versus infant skin. Pink color indicates significantly higher gene expression in adult skin versus infant samples. If multiple probes matched to same gene, the one with smallest P value was selected to color the box.
(TIF)

S6 Fig KEGG pathway map for antigen processing and presentation that was increased in adults. Blue color indicates significantly lower gene expression in adult skin versus infant skin. Pink color indicates significantly higher gene expression in adult skin versus infant samples. If multiple probes matched to same gene, the one with smallest P value was selected to color the box.
(TIF)

S1 Table Top Gene Ontology themes increased for infants versus adults with adjusted p value < = 0.0001. (DOCX)

S2 Table Top Gene Ontology themes increased for adults versus with adjusted p value < = 0.0001. (DOCX)

S1 Document Validation of transcriptomics data. (DOCX)

S1 File Gene expression data. (XLSX)

## References

[1] Hoath S, Pickens W. The Biology of Vernix. In: Hoath SB, Maibach H, editors. Neonatal Skin: Structure and Function. 2nd ed. New York: Marcel Dekker; 2003. p. 193–210.

[2] Kimball AB, Alora-Palli MB, Tamura M, Mullins LA, Soh C, Binder RL, et al. Age-induced and photoinduced changes in gene expression profiles in facial skin of Caucasian females across 6 decades of age. J Am Acad Dermatol. 2018;78(1):29–39 e7. Epub 2017/11/18. doi: 10.1016/j.jaad.2017.09.012 . 29146147

[3] Kottner J, Lichterfeld A, Blume-Peytavi U. Maintaining skin integrity in the aged: a systematic review. Br J Dermatol. 2013;169(3):528–42. Epub 2013/06/19. doi: 10.1111/bjd.12469 . 23773110

[4] Cunico RL, Maibach HI, Khan H, Bloom E. Skin barrier properties in the newborn. Transepidermal water loss and carbon dioxide emission rates. Biol Neonate. 1977;32(3–4):177–82. doi: 10.1159/000241013 . 603802

[5] Yosipovitch G, Maayan-Metzger A, Merlob P, Sirota L. Skin barrier properties in different body areas in neonates. Pediatrics. 2000;106(1 Pt 1):105–8. Epub 2000/07/06. doi: 10.1542/peds.106.1.105 . 10878157

[6] Stamatas GN, Nikolovski J, Luedtke MA, Kollias N, Wiegand BC. Infant skin microstructure assessed in vivo differs from adult skin in organization and at the cellular level. Pediatr Dermatol. 2010;27(2):125–31. doi: 10.1111/j.1525-1470.2009.00973.x . 19804498

[7] Visscher MO, Chatterjee R, Munson KA, Pickens WL, Hoath SB. Changes in diapered and nondiapered infant skin over the first month of life. Pediatr Dermatol. 2000;17(1):45–51. Epub 2000/03/18. pde1711 [pii]. doi: 10.1046/j.1525-1470.2000.01711.x . 10720988

[8] Fluhr JW, Darlenski R, Lachmann N, Baudouin C, Msika P, De Belilovsky C, et al. Infant epidermal skin physiology: adaptation after birth. Br J Dermatol. 2012;166(3):483–90. Epub 2011/10/05. doi: 10.1111/j.1365-2133.2011.10659.x . 21967466

[9] Rippke F, Schreiner V, Schwanitz HJ. The acidic milieu of the horny layer: new findings on the physiology and pathophysiology of skin pH. Am J Clin Dermatol. 2002;3(4):261–72. Epub 2002/05/16. 030404 [pii]. doi: 10.2165/00128071-200203040-00004 . 12010071

[10] Schmid-Wendtner MH, Korting HC. The pH of the skin surface and its impact on the barrier function. Skin Pharmacol Physiol. 2006;19(6):296–302. Epub 2006/07/26. 94670 [pii] doi: 10.1159/000094670 . 16864974

[11] Elias PM. The how, why and clinical importance of stratum corneum acidification. Exp Dermatol. 2017;26(11):999–1003. Epub 2017/03/08. doi: 10.1111/exd.13329 . 28266738

[12] Gallo RL. Human Skin Is the Largest Epithelial Surface for Interaction with Microbes. J Invest Dermatol. 2017;137(6):1213–4. Epub 2017/04/12. doi: 10.1016/j.jid.2016.11.045 ; PubMed Central PMCID: PMC5814118. 28395897 PMC5814118

[13] Naik S, Bouladoux N, Wilhelm C, Molloy MJ, Salcedo R, Kastenmuller W, et al. Compartmentalized control of skin immunity by resident commensals. Science. 2012;337(6098):1115–9. Epub 2012/07/28. doi: 10.1126/science.1225152 ; PubMed Central PMCID: PMC3513834. 22837383 PMC3513834

[14] Chen YE, Fischbach MA, Belkaid Y. Erratum: Skin microbiota-host interactions. Nature. 2018;555(7697):543. Epub 2018/03/23. doi: 10.1038/nature25994 . 29565366

[15] Chen YE, Fischbach MA, Belkaid Y. Skin microbiota-host interactions. Nature. 2018;553(7689):427–36. Epub 2018/01/25. doi: 10.1038/nature25177 ; PubMed Central PMCID: PMC6075667. 29364286 PMC6075667

[16] Elias PM. The skin barrier as an innate immune element. Semin Immunopathol. 2007;29(1):3–14. doi: 10.1007/s00281-007-0060-9 . 17621950

[17] Baurecht H, Ruhlemann MC, Rodriguez E, Thielking F, Harder I, Erkens AS, et al. Epidermal lipid composition, barrier integrity, and eczematous inflammation are associated with skin microbiome configuration. J Allergy Clin Immunol. 2018;141(5):1668–76 e16. Epub 2018/02/09. doi: 10.1016/j.jaci.2018.01.019 . 29421277

[18] Pammi M, O’Brien JL, Ajami NJ, Wong MC, Versalovic J, Petrosino JF. Development of the cutaneous microbiome in the preterm infant: A prospective longitudinal study. PloS one. 2017;12(4):e0176669. doi: 10.1371/journal.pone.0176669 ; PubMed Central PMCID: PMC5407830. 28448623 PMC5407830

[19] Visscher MO, Carr AN, Winget J, Huggins T, Bascom CC, Isfort R, et al. Biomarkers of neonatal skin barrier adaptation reveal substantial differences compared to adult skin. Pediatric Research. 2020. doi: 10.1038/s41390-020-1035-y 32599611 PMC8119241

[20] Hu T, Khambatta ZS, Hayden PJ, Bolmarcich J, Binder RL, Robinson MK, et al. Xenobiotic metabolism gene expression in the EpiDermin vitro 3D human epidermis model compared to human skin. Toxicol In Vitro. 2010;24(5):1450–63. Epub 2010/03/31. doi: 10.1016/j.tiv.2010.03.013 . 20350595

[21] Naciff JM, Jump ML, Torontali SM, Carr GJ, Tiesman JP, Overmann GJ, et al. Gene expression profile induced by 17alpha-ethynyl estradiol, bisphenol A, and genistein in the developing female reproductive system of the rat. Toxicol Sci. 2002;68(1):184–99. Epub 2002/06/21. doi: 10.1093/toxsci/68.1.184 . 12075121

[22] Naciff JM, Khambatta ZS, Carr GJ, Tiesman JP, Singleton DW, Khan SA, et al. Dose- and Time-Dependent Transcriptional Response of Ishikawa Cells Exposed to Genistein. Toxicol Sci. 2016;151(1):71–87. Epub 2016/02/13. doi: 10.1093/toxsci/kfw024 ; PubMed Central PMCID: PMC4914796. 26865667 PMC4914796

[23] Naciff JM, Khambatta ZS, Thomason RG, Carr GJ, Tiesman JP, Singleton DW, et al. The genomic response of a human uterine endometrial adenocarcinoma cell line to 17alpha-ethynyl estradiol. Toxicol Sci. 2009;107(1):40–55. Epub 2008/10/22. doi: 10.1093/toxsci/kfn219 . 18936297

[24] Yu G, Wang LG, Han Y, He QY. clusterProfiler: an R package for comparing biological themes among gene clusters. OMICS. 2012;16(5):284–7. Epub 2012/03/30. doi: 10.1089/omi.2011.0118 ; PubMed Central PMCID: PMC3339379. 22455463 PMC3339379

[25] Szklarczyk D, Gable AL, Lyon D, Junge A, Wyder S, Huerta-Cepas J, et al. STRING v11: protein-protein association networks with increased coverage, supporting functional discovery in genome-wide experimental datasets. Nucleic Acids Res. 2019;47(D1):D607–D13. Epub 2018/11/27. doi: 10.1093/nar/gky1131 ; PubMed Central PMCID: PMC6323986. 30476243 PMC6323986

[26] Raudvere U, Kolberg L, Kuzmin I, Arak T, Adler P, Peterson H, et al. g:Profiler: a web server for functional enrichment analysis and conversions of gene lists (2019 update). Nucleic Acids Res. 2019;47(W1):W191–W8. Epub 2019/05/09. doi: 10.1093/nar/gkz369 ; PubMed Central PMCID: PMC6602461. 31066453 PMC6602461

[27] Merico D, Isserlin R, Stueker O, Emili A, Bader GD. Enrichment map: a network-based method for gene-set enrichment visualization and interpretation. PloS one. 2010;5(11):e13984. Epub 2010/11/19. doi: 10.1371/journal.pone.0013984 ; PubMed Central PMCID: PMC2981572. 21085593 PMC2981572

[28] Supek F, Bosnjak M, Skunca N, Smuc T. REVIGO summarizes and visualizes long lists of gene ontology terms. PloS one. 2011;6(7):e21800. Epub 2011/07/27. doi: 10.1371/journal.pone.0021800 ; PubMed Central PMCID: PMC3138752. 21789182 PMC3138752

[29] Gu Z, Eils R, Schlesner M. Complex heatmaps reveal patterns and correlations in multidimensional genomic data. Bioinformatics. 2016;32(18):2847–9. Epub 2016/05/22. doi: 10.1093/bioinformatics/btw313 . 27207943

[30] Karoulias SZ, Taye N, Stanley S, Hubmacher D. The ADAMTS/Fibrillin Connection: Insights into the Biological Functions of ADAMTS10 and ADAMTS17 and Their Respective Sister Proteases. Biomolecules. 2020;10(4). Epub 2020/04/16. doi: 10.3390/biom10040596 ; PubMed Central PMCID: PMC7226509. 32290605 PMC7226509

[31] Khan I, Maldonado E, Vasconcelos V, O’Brien SJ, Johnson WE, Antunes A. Mammalian keratin associated proteins (KRTAPs) subgenomes: disentangling hair diversity and adaptation to terrestrial and aquatic environments. BMC Genomics. 2014;15:779. Epub 2014/09/12. doi: 10.1186/1471-2164-15-779 ; PubMed Central PMCID: PMC4180150. 25208914 PMC4180150

[32] Hoeger PH, Enzmann CC. Skin physiology of the neonate and young infant: a prospective study of functional skin parameters during early infancy. Pediatr Dermatol. 2002;19(3):256–62. doi: 10.1046/j.1525-1470.2002.00082.x . 12047648

[33] Nikolovski J, Stamatas GN, Kollias N, Wiegand BC. Barrier function and water-holding and transport properties of infant stratum corneum are different from adult and continue to develop through the first year of life. J Invest Dermatol. 2008;128(7):1728–36. Epub 2008/01/18. 5701239 [pii] doi: 10.1038/sj.jid.5701239 . 18200056

[34] Westerberg R, Tvrdik P, Unden AB, Mansson JE, Norlen L, Jakobsson A, et al. Role for ELOVL3 and fatty acid chain length in development of hair and skin function. J Biol Chem. 2004;279(7):5621–9. Epub 2003/10/29. doi: 10.1074/jbc.M310529200 . 14581464

[35] Wang Z, Wang DH, Park HG, Yan Y, Goykhman Y, Lawrence P, et al. Identification of genes mediating branched chain fatty acid elongation. FEBS Lett. 2019;593(14):1807–17. Epub 2019/05/23. doi: 10.1002/1873-3468.13451 . 31116414

[36] Berdyshev E, Goleva E, Bronova I, Dyjack N, Rios C, Jung J, et al. Lipid abnormalities in atopic skin are driven by type 2 cytokines. JCI Insight. 2018;3(4). Epub 2018/02/23. doi: 10.1172/jci.insight.98006 ; PubMed Central PMCID: PMC5916244. 29467325 PMC5916244

[37] Daley WP, Peters SB, Larsen M. Extracellular matrix dynamics in development and regenerative medicine. J Cell Sci. 2008;121(Pt 3):255–64. Epub 2008/01/25. doi: 10.1242/jcs.006064 . 18216330

[38] Sumigray KD, Lechler T. Cell adhesion in epidermal development and barrier formation. Curr Top Dev Biol. 2015;112:383–414. Epub 2015/03/04. doi: 10.1016/bs.ctdb.2014.11.027 ; PubMed Central PMCID: PMC4737682. 25733147 PMC4737682

[39] Mauch C, Zamek J, Abety AN, Grimberg G, Fox JW, Zigrino P. Accelerated wound repair in ADAM-9 knockout animals. J Invest Dermatol. 2010;130(8):2120–30. Epub 2010/04/09. doi: 10.1038/jid.2010.60 . 20376065

[40] Gunther W, Skaftnesmo KO, Arnold H, Bjerkvig R, Terzis AJ. Distribution patterns of the anti-angiogenic protein ADAMTS-1 during rat development. Acta histochemica. 2005;107(2):121–31. Epub 2005/05/10. doi: 10.1016/j.acthis.2004.07.009 . 15878613

[41] Thomson J, Singh M, Eckersley A, Cain SA, Sherratt MJ, Baldock C. Fibrillin microfibrils and elastic fibre proteins: Functional interactions and extracellular regulation of growth factors. Semin Cell Dev Biol. 2019;89:109–17. Epub 2018/07/18. doi: 10.1016/j.semcdb.2018.07.016 ; PubMed Central PMCID: PMC6461133. 30016650 PMC6461133

[42] Hu MS, Borrelli MR, Hong WX, Malhotra S, Cheung ATM, Ransom RC, et al. Embryonic skin development and repair. Organogenesis. 2018;14(1):46–63. Epub 2018/02/09. doi: 10.1080/15476278.2017.1421882 ; PubMed Central PMCID: PMC6150059. 29420124 PMC6150059

[43] Barrientos S, Stojadinovic O, Golinko MS, Brem H, Tomic-Canic M. Growth factors and cytokines in wound healing. Wound Repair Regen. 2008;16(5):585–601. Epub 2009/01/09. doi: 10.1111/j.1524-475X.2008.00410.x . 19128254

[44] Caniggia I, Mostachfi H, Winter J, Gassmann M, Lye SJ, Kuliszewski M, et al. Hypoxia-inducible factor-1 mediates the biological effects of oxygen on human trophoblast differentiation through TGFbeta(3). J Clin Invest. 2000;105(5):577–87. Epub 2000/03/11. doi: 10.1172/JCI8316 ; PubMed Central PMCID: PMC289179. 10712429 PMC289179

[45] Coolen NA, Schouten KC, Middelkoop E, Ulrich MM. Comparison between human fetal and adult skin. Arch Dermatol Res. 2010;302(1):47–55. Epub 2009/08/25. doi: 10.1007/s00403-009-0989-8 ; PubMed Central PMCID: PMC2799629. 19701759 PMC2799629

[46] Simon AK, Hollander GA, McMichael A. Evolution of the immune system in humans from infancy to old age. Proc Biol Sci. 2015;282(1821):20143085. Epub 2015/12/25. doi: 10.1098/rspb.2014.3085 ; PubMed Central PMCID: PMC4707740. 26702035 PMC4707740

[47] Levy O. Innate immunity of the newborn: basic mechanisms and clinical correlates. Nat Rev Immunol. 2007;7(5):379–90. Epub 2007/04/26. doi: 10.1038/nri2075 [pii] 10.1038/nri2075 [doi]. . 17457344

[48] Tomlin H, Piccinini AM. A complex interplay between the extracellular matrix and the innate immune response to microbial pathogens. Immunology. 2018;155(2):186–201. Epub 2018/06/17. doi: 10.1111/imm.12972 ; PubMed Central PMCID: PMC6142291. 29908065 PMC6142291

[49] Arora S, Gordon J, Hook M. Collagen Binding Proteins of Gram-Positive Pathogens. Front Microbiol. 2021;12:628798. Epub 2021/02/23. doi: 10.3389/fmicb.2021.628798 ; PubMed Central PMCID: PMC7893114. 33613497 PMC7893114

[50] Bhattacharjee O, Ayyangar U, Kurbet AS, Ashok D, Raghavan S. Unraveling the ECM-Immune Cell Crosstalk in Skin Diseases. Front Cell Dev Biol. 2019;7:68. Epub 2019/05/28. doi: 10.3389/fcell.2019.00068 ; PubMed Central PMCID: PMC6514232. 31134198 PMC6514232

[51] Xu J, Spitale RC, Guan L, Flynn RA, Torre EA, Li R, et al. Novel Gene Expression Profile of Women with Intrinsic Skin Youthfulness by Whole Transcriptome Sequencing. PloS one. 2016;11(11):e0165913. Epub 2016/11/10. doi: 10.1371/journal.pone.0165913 ; PubMed Central PMCID: PMC5102383 not alter our adherence to PLOS ONE policies on sharing data and materials. 27829007 PMC5102383

[52] Ubags ND, Trompette A, Pernot J, Nibbering B, Wong NC, Pattaroni C, et al. Microbiome-induced antigen-presenting cell recruitment coordinates skin and lung allergic inflammation. J Allergy Clin Immunol. 2020. Epub 2020/07/18. doi: 10.1016/j.jaci.2020.06.030 32679208

[53] Geng Z, Wang J, Pan L, Li M, Zhang J, Cai X, et al. Microarray Analysis of Differential Gene Expression Profile Between Human Fetal and Adult Heart. Pediatr Cardiol. 2017;38(4):700–6. Epub 2017/03/24. doi: 10.1007/s00246-017-1569-x . 28331934

[54] Colaco CA, Bailey CR, Walker KB, Keeble J. Heat shock proteins: stimulators of innate and acquired immunity. BioMed research international. 2013;2013:461230. Epub 2013/06/14. doi: 10.1155/2013/461230 ; PubMed Central PMCID: PMC3677648. 23762847 PMC3677648

[55] Binder RJ. Hsp receptors: the cases of identity and mistaken identity. Curr Opin Mol Ther. 2009;11(1):62–71. Epub 2009/01/27. . 19169961

[56] Friedland JS, Shattock R, Remick DG, Griffin GE. Mycobacterial 65-kD heat shock protein induces release of proinflammatory cytokines from human monocytic cells. Clin Exp Immunol. 1993;91(1):58–62. Epub 1993/01/01. doi: 10.1111/j.1365-2249.1993.tb03354.x PubMed Central PMCID: PMC1554637. 8419086 PMC1554637

[57] Scieglinska D, Krawczyk Z, Sojka DR, Gogler-Piglowska A. Heat shock proteins in the physiology and pathophysiology of epidermal keratinocytes. Cell Stress Chaperones. 2019;24(6):1027–44. Epub 2019/11/18. doi: 10.1007/s12192-019-01044-5 ; PubMed Central PMCID: PMC6882751. 31734893 PMC6882751

[58] Sun BK, Boxer LD, Ransohoff JD, Siprashvili Z, Qu K, Lopez-Pajares V, et al. CALML5 is a ZNF750- and TINCR-induced protein that binds stratifin to regulate epidermal differentiation. Genes Dev. 2015;29(21):2225–30. Epub 2015/11/08. doi: 10.1101/gad.267708.115 ; PubMed Central PMCID: PMC4647556. 26545810 PMC4647556

[59] Brunner PM, Israel A, Zhang N, Leonard A, Wen HC, Huynh T, et al. Early-onset pediatric atopic dermatitis is characterized by TH2/TH17/TH22-centered inflammation and lipid alterations. J Allergy Clin Immunol. 2018;141(6):2094–106. Epub 2018/05/08. doi: 10.1016/j.jaci.2018.02.040 . 29731129

[60] Lener T, Moll PR, Rinnerthaler M, Bauer J, Aberger F, Richter K. Expression profiling of aging in the human skin. Exp Gerontol. 2006;41(4):387–97. Epub 2006/03/15. doi: 10.1016/j.exger.2006.01.012 . 16530368

[61] Biagini Myers JM, Sherenian MG, Baatyrbek Kyzy A, Alarcon R, An A, Flege Z, et al. Events in Normal Skin Promote Early-Life Atopic Dermatitis-The MPAACH Cohort. J Allergy Clin Immunol Pract. 2020;8(7):2285–93 e6. Epub 2020/04/18. doi: 10.1016/j.jaip.2020.03.048 ; PubMed Central PMCID: PMC7338239. 32302785 PMC7338239

[62] Brunner PM, Israel A, Leonard A, Pavel AB, Kim HJ, Zhang N, et al. Distinct transcriptomic profiles of early-onset atopic dermatitis in blood and skin of pediatric patients. Ann Allergy Asthma Immunol. 2019;122(3):318–30 e3. Epub 2018/12/07. doi: 10.1016/j.anai.2018.11.025 . 30508584

[63] McAleer MA, Jakasa I, Raj N, O’Donnell CPF, Lane ME, Rawlings AV, et al. Early-life regional and temporal variation in filaggrin-derived natural moisturizing factor, filaggrin-processing enzyme activity, corneocyte phenotypes and plasmin activity: implications for atopic dermatitis. Br J Dermatol. 2018;179(2):431–41. Epub 2018/04/25. doi: 10.1111/bjd.16691 ; PubMed Central PMCID: PMC6175251. 29691836 PMC6175251

[64] McClanahan D, Wong A, Kezic S, Samrao A, Hajar T, Hill E, et al. A randomized controlled trial of an emollient with ceramide and filaggrin-associated amino acids for the primary prevention of atopic dermatitis in high-risk infants. J Eur Acad Dermatol Venereol. 2019;33(11):2087–94. Epub 2019/07/10. doi: 10.1111/jdv.15786 . 31287580

[65] Li J, Zheng L, Uchiyama A, Bin L, Mauro TM, Elias PM, et al. A data mining paradigm for identifying key factors in biological processes using gene expression data. Scientific reports. 2018;8(1):9083. Epub 2018/06/15. doi: 10.1038/s41598-018-27258-8 ; PubMed Central PMCID: PMC5998123. 29899432 PMC5998123

