# Supplementary figures and images for: Newborn infant skin gene expression: Remarkable differences versus adults
Source: PLoS One. 2021 Oct 19;16(10):e0258554. doi: 10.1371/journal.pone.0258554 (PMC8525758; doi:10.1371/journal.pone.0258554)

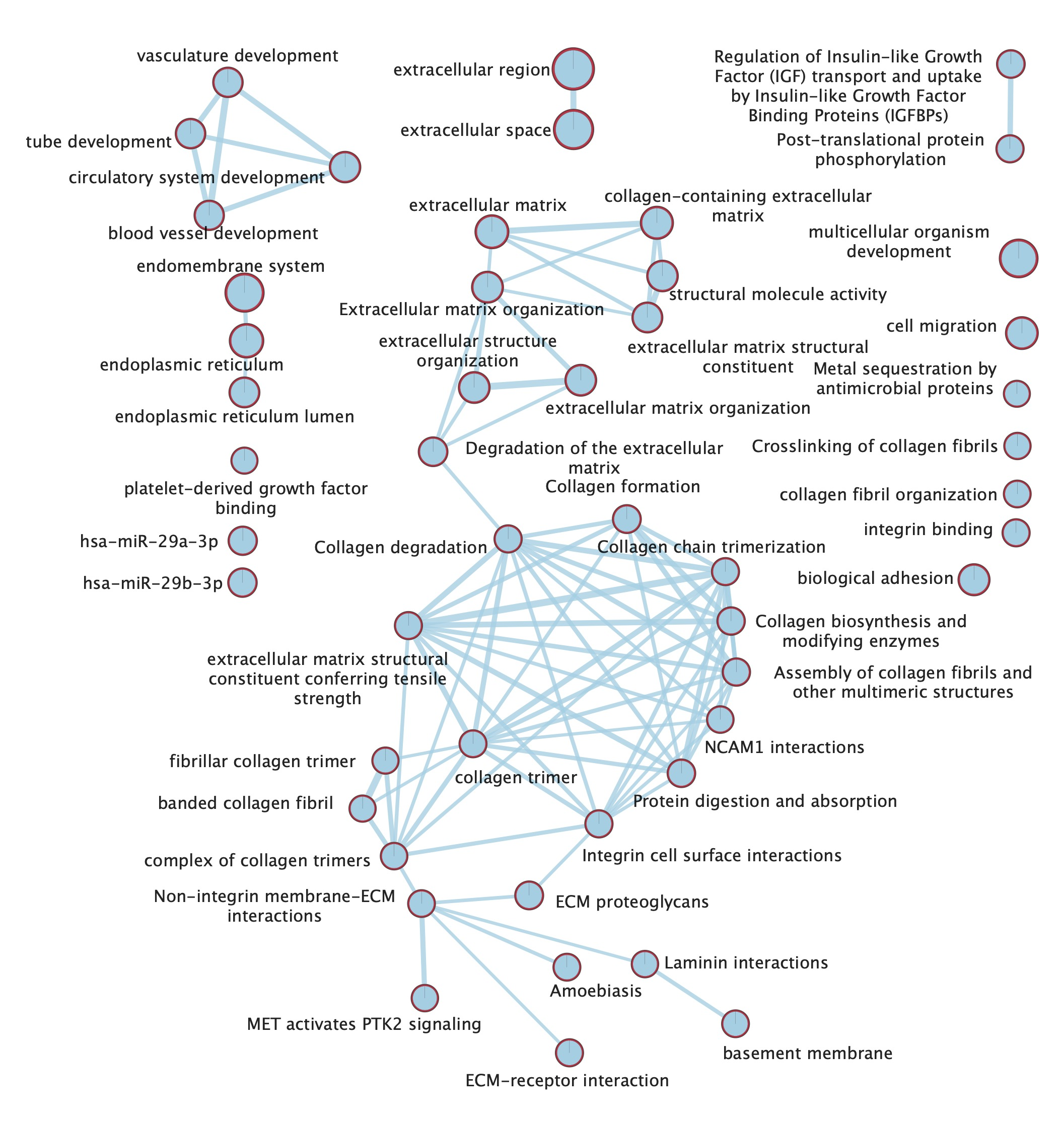

Supplement: S1 Fig — Significant biological themes from G:Profiler analysis with adjusted p value <0.00001 were displayed in nodes. Edges were shown with similarity > = 0.5 between two nodes. (TIF) [file pone.0258554.s001.tif]

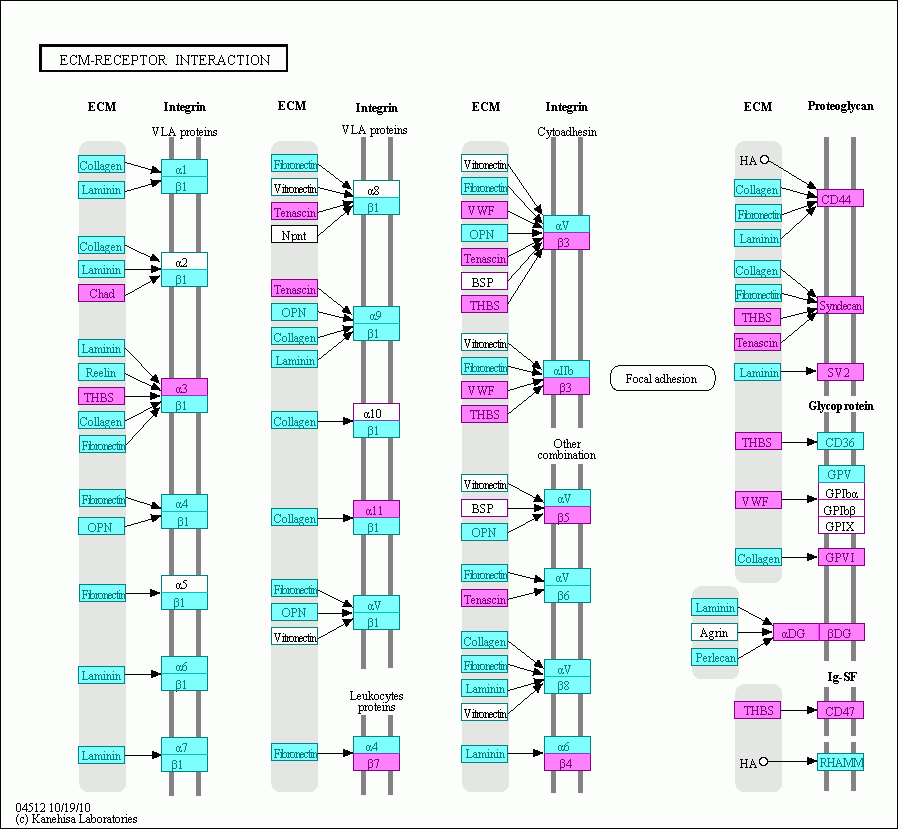

Supplement: S2 Fig — Blue color indicates significantly lower gene expression in adult skin versus infant skin. Pink color indicates significantly higher gene expression in adult skin versus infant samples. If multiple probes matched to the same gene, the one with smallest p value was selected to color the box. (TIF) [file pone.0258554.s002.tif]

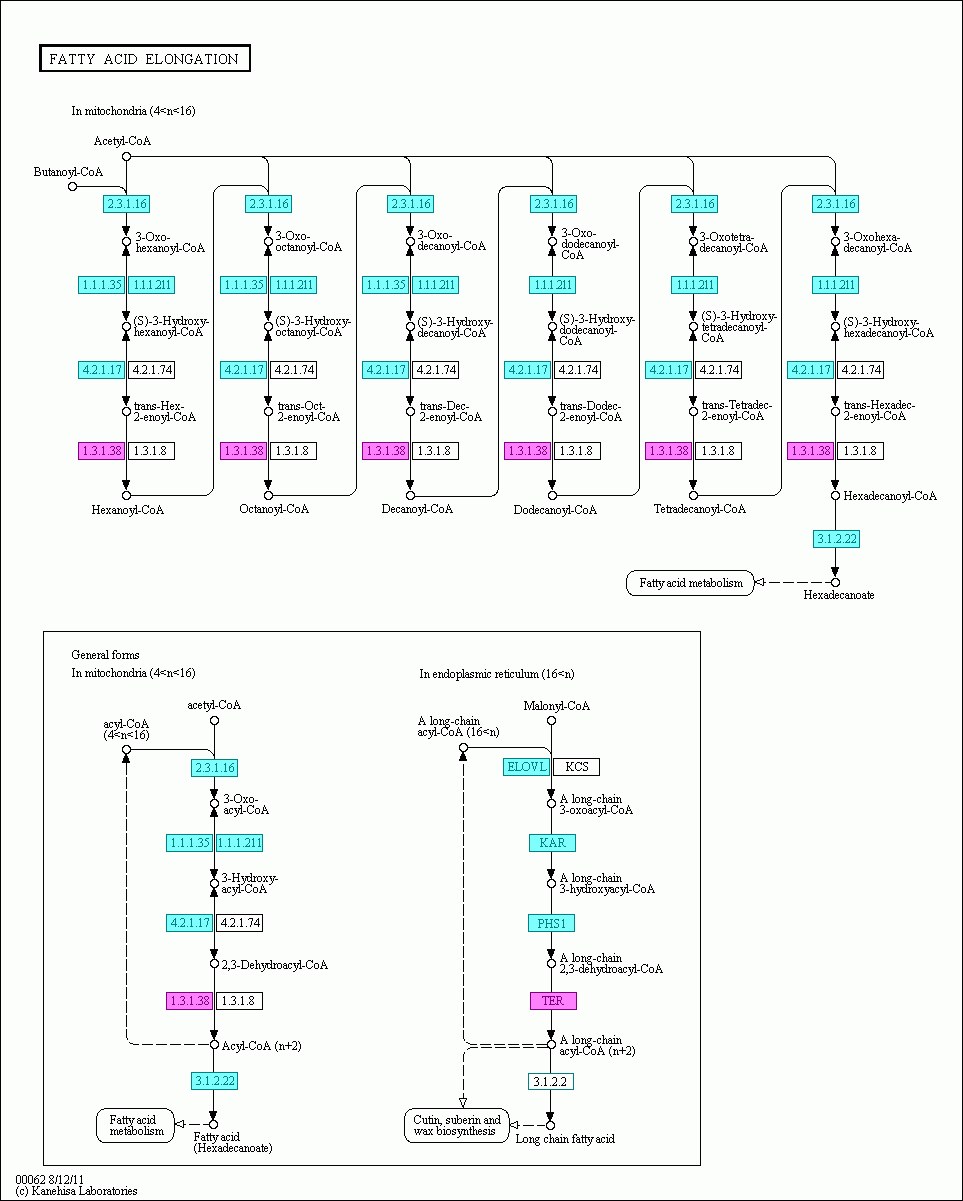

Supplement: S3 Fig — Blue color indicates significantly lower gene expression in adult skin versus infant skin. Pink color indicates significantly higher gene expression in adult skin versus infant samples. If multiple probes matched to same gene, the one with smallest P value was selected to color the box. (TIF) [file pone.0258554.s003.tif]

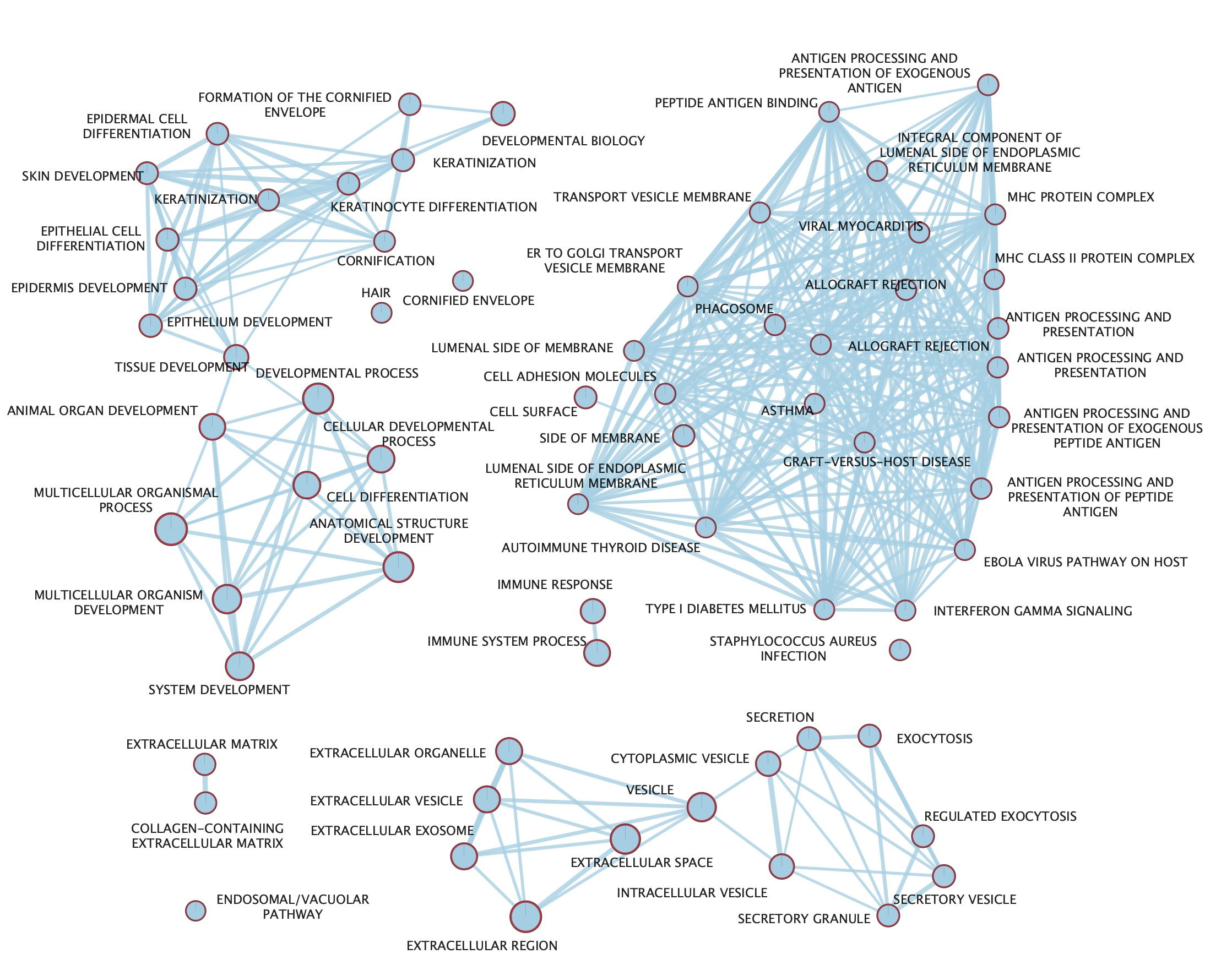

Supplement: S4 Fig — Significant GO themes of biological processes from G:Profiler analysis with adjusted P value <0.00001 were displayed in nodes. Edges were shown with similarity > = 0.5 between two nodes. (TIF) [file pone.0258554.s004.tif]

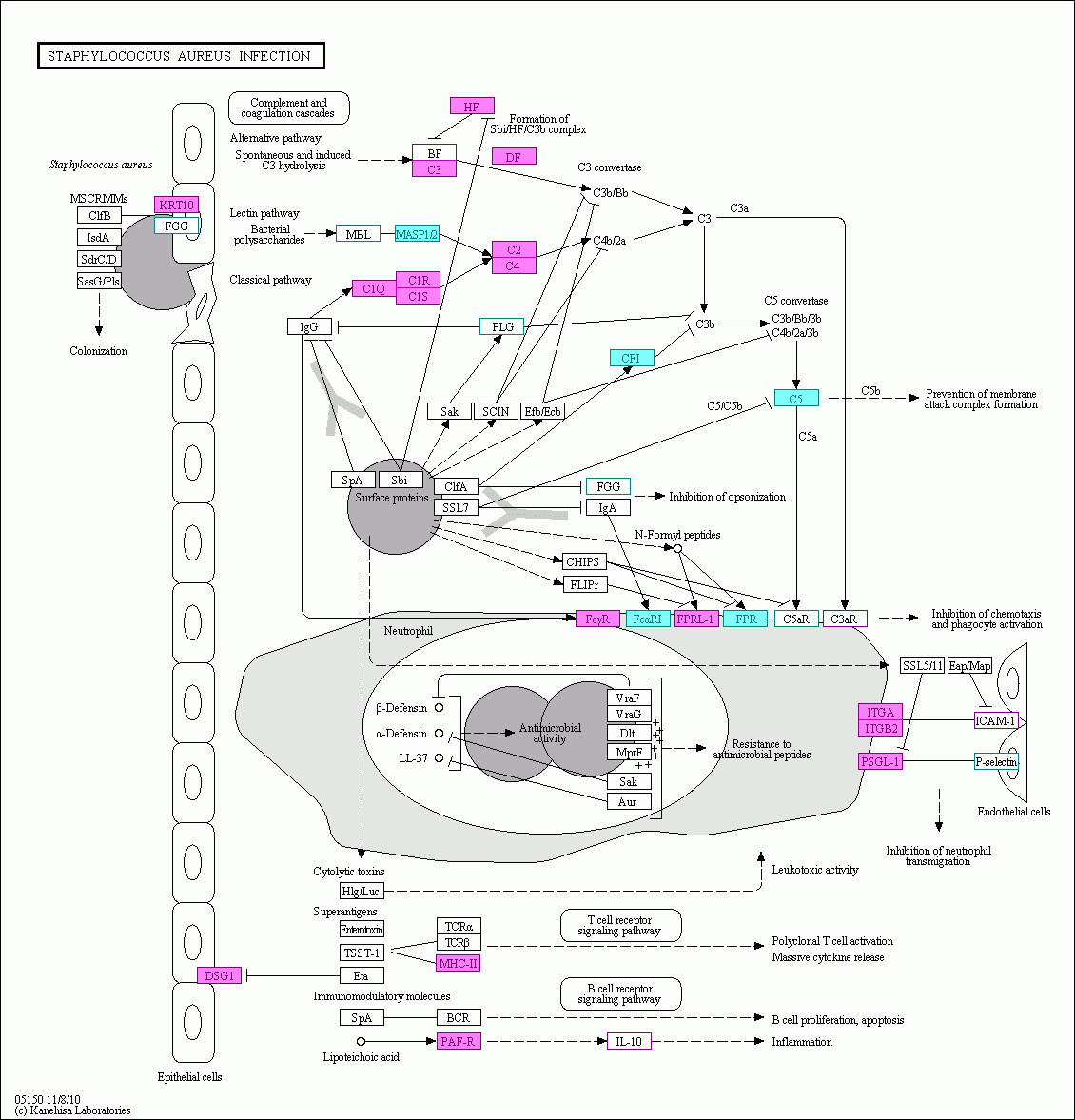

Supplement: S5 Fig — Blue color indicates significantly lower gene expression in adult skin versus infant skin. Pink color indicates significantly higher gene expression in adult skin versus infant samples. If multiple probes matched to same gene, the one with smallest P value was selected to color the box. (TIF) [file pone.0258554.s005.tif]

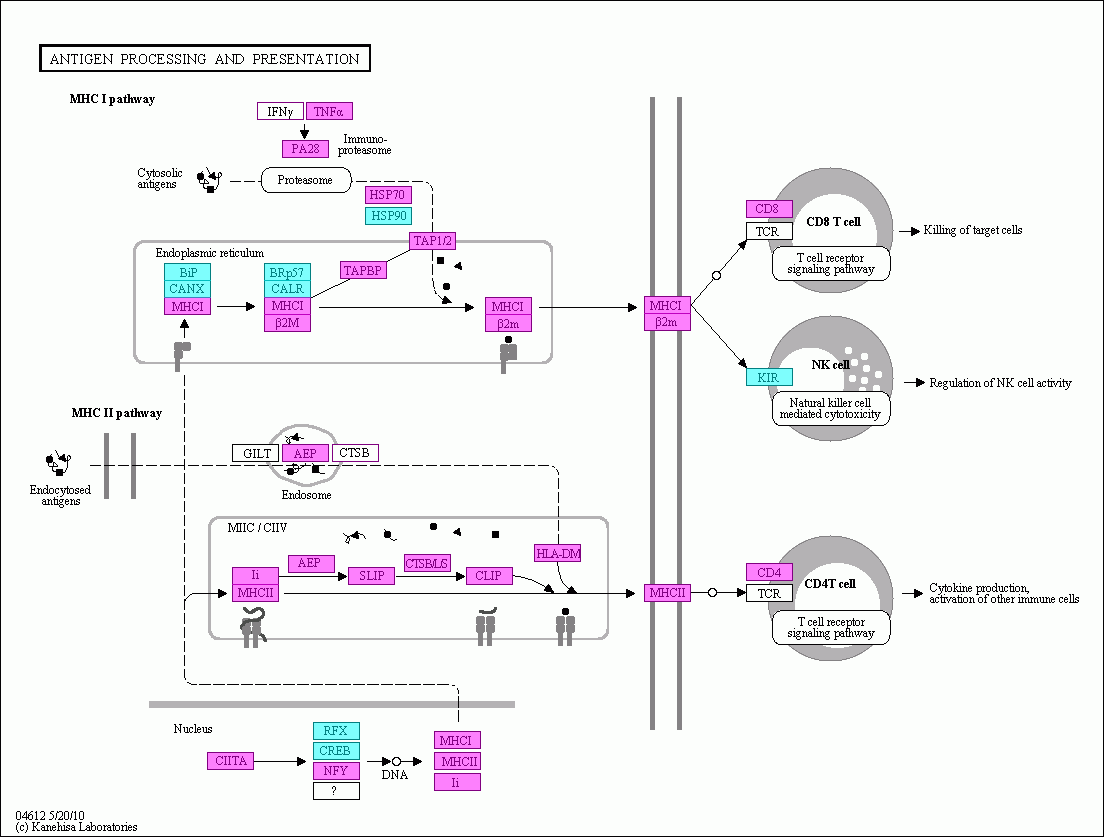

Supplement: S6 Fig — Blue color indicates significantly lower gene expression in adult skin versus infant skin. Pink color indicates significantly higher gene expression in adult skin versus infant samples. If multiple probes matched to same gene, the one with smallest P value was selected to color the box. (TIF) [file pone.0258554.s006.tif]
