# Supplementary material for: Newborn infant skin gene expression: Remarkable differences versus adults
Source: PLoS One. 2021 Oct 19;16(10):e0258554. doi: 10.1371/journal.pone.0258554 (PMC8525758; doi:10.1371/journal.pone.0258554)
Supplement: S1 Table — (DOCX) [file pone.0258554.s007.docx]

**Supplementary Table S1.** Top Gene Ontology themes increased for infants versus adults with adjusted p value <=0.0001

| **ID** | **Description** | **Count** | **pvalue** | **p.adjust** | **GO**  **Category** | **Expression** |
| --- | --- | --- | --- | --- | --- | --- |
| GO:0030198 | extracellular matrix organization | 44 | 1.05E-27 | 2.37E-24 | BP | Infant>Adult |
| GO:0043062 | extracellular structure organization | 44 | 1.18E-27 | 2.37E-24 | BP | Infant>Adult |
| GO:0001568 | blood vessel development | 38 | 7.19E-13 | 9.61E-10 | BP | Infant>Adult |
| GO:0001944 | vasculature development | 38 | 2.3E-12 | 2.31E-09 | BP | Infant>Adult |
| GO:0072358 | cardiovascular system development | 38 | 3.3E-12 | 2.65E-09 | BP | Infant>Adult |
| GO:0030199 | collagen fibril organization | 12 | 1.19E-11 | 7.97E-09 | BP | Infant>Adult |
| GO:0048514 | blood vessel morphogenesis | 31 | 7.54E-10 | 4.32E-07 | BP | Infant>Adult |
| GO:0033993 | response to lipid | 39 | 1.62E-09 | 6.5E-07 | BP | Infant>Adult |
| GO:0035239 | tube morphogenesis | 37 | 1.55E-09 | 6.5E-07 | BP | Infant>Adult |
| GO:0035295 | tube development | 42 | 1.36E-09 | 6.5E-07 | BP | Infant>Adult |
| GO:0001501 | skeletal system development | 28 | 4.47E-09 | 1.63E-06 | BP | Infant>Adult |
| GO:0032963 | collagen metabolic process | 13 | 5.19E-09 | 1.74E-06 | BP | Infant>Adult |
| GO:0001101 | response to acid chemical | 22 | 7.89E-09 | 2.44E-06 | BP | Infant>Adult |
| GO:0070848 | response to growth factor | 31 | 4.12E-08 | 1.18E-05 | BP | Infant>Adult |
| GO:0071363 | cellular response to growth factor stimulus | 30 | 5.41E-08 | 1.45E-05 | BP | Infant>Adult |
| GO:0001503 | ossification | 22 | 7.43E-08 | 1.86E-05 | BP | Infant>Adult |
| GO:0044272 | sulfur compound biosynthetic process | 15 | 8.72E-08 | 2.06E-05 | BP | Infant>Adult |
| GO:0006631 | fatty acid metabolic process | 21 | 9.7E-08 | 2.16E-05 | BP | Infant>Adult |
| GO:0032787 | monocarboxylic acid metabolic process | 28 | 1.78E-07 | 3.75E-05 | BP | Infant>Adult |
| GO:0060348 | bone development | 16 | 1.98E-07 | 3.97E-05 | BP | Infant>Adult |
| GO:0001525 | angiogenesis | 24 | 3.4E-07 | 6.49E-05 | BP | Infant>Adult |
| GO:0071560 | cellular response to transforming growth factor beta stimulus | 16 | 4.41E-07 | 8.04E-05 | BP | Infant>Adult |
| GO:0031012 | extracellular matrix | 62 | 9.11E-39 | 3.37E-36 | CC | Infant>Adult |
| GO:0062023 | collagen-containing extracellular matrix | 52 | 4.38E-34 | 8.1E-32 | CC | Infant>Adult |
| GO:0005788 | endoplasmic reticulum lumen | 34 | 2.9E-20 | 3.57E-18 | CC | Infant>Adult |
| GO:0005581 | collagen trimer | 19 | 1.07E-17 | 9.92E-16 | CC | Infant>Adult |
| GO:0098644 | complex of collagen trimers | 9 | 2.86E-12 | 2.12E-10 | CC | Infant>Adult |
| GO:0005604 | basement membrane | 15 | 8.71E-12 | 5.37E-10 | CC | Infant>Adult |
| GO:0005583 | fibrillar collagen trimer | 6 | 4.84E-09 | 2.24E-07 | CC | Infant>Adult |
| GO:0098643 | banded collagen fibril | 6 | 4.84E-09 | 2.24E-07 | CC | Infant>Adult |
| GO:0005201 | extracellular matrix structural constituent | 37 | 1.4E-33 | 8.34E-31 | MF | Infant>Adult |
| GO:0005198 | structural molecule activity | 43 | 2.47E-16 | 7.37E-14 | MF | Infant>Adult |
| GO:0030020 | extracellular matrix structural constituent conferring tensile strength | 14 | 5.86E-16 | 1.17E-13 | MF | Infant>Adult |
| GO:0048407 | platelet-derived growth factor binding | 5 | 3.33E-07 | 4.96E-05 | MF | Infant>Adult |
