# Supplementary material for: Newborn infant skin gene expression: Remarkable differences versus adults
Source: PLoS One. 2021 Oct 19;16(10):e0258554. doi: 10.1371/journal.pone.0258554 (PMC8525758; doi:10.1371/journal.pone.0258554)
Supplement: S1 Document — (DOCX) [file pone.0258554.s009.docx]

Supplementary Document (S1 Document)

**Validation of Transcriptomics Data**

The Procter & Gamble genomics laboratory was established in 1999 and, from its inception, sought to develop rigorous, industrialized quality control protocols for the execution of GeneChip experiments for genomics needs company-wide. Established IQ/OQ/PQ protocols from the P&G QA organization were used to establish and maintain the laboratory equipment. Before, any experimental samples were run, multiple studies utilizing over 100 GeneChips were designed by P&G team consisting of biologists, statisticians, project engineers, and bioinformaticists. In addition to using specific host cells and tissues, in vitro transcribed RNA transcripts were used to evaluate the quantitative capacity of these GeneChip experiments and several studies were validated using quantitative RT-PCR and ddPCR (unpublished data). These control experiments were designed to identify sources of bias and experimental variability and to determine optimal sample sizes for prospective experiments and to fine-tune statistical and bioinformatics analyses.

All project-based experiments are designed with these quality control methods in place by the experimental design team consisting of a core group of genomics, statistics, bioinformatics personnel as well as project leaders and domain experts. This design team has control over the experiment and establish protocols and acceptance criteria before any samples are collected or processed. They also supervise quality at key milestones of the experiment, including Sample Extraction, RNA Isolation, Target Preparation, GeneChip Processing, Statistical Analysis, and Bioinformatics Analysis.

To date, over 1,000 studies and over 150,000 genomics samples have been processed using these rigorous QC metrics.

The data from all genomics samples undergo rigorous quality control procedure to detect potential outliers due to processing, instrumentation, or other reasons. This process includes, among others, examination of the GeneChip level Affymetrix QC metrics (Affymetrix): Raw Q, Scaling Factor, Noise Average, and Background Average. Probe set level QC metrics, such as Prediction Interval Analysis, Principal Components Plots, and Pairs Plots, as well as Leave-One-Out analyses were also performed to ensure high quality data.

As the present study was a pathway identification analysis rather than a biomarker generation analysis, quantitative RT-PCR was not performed. In our experience, the size of the study, the overall study design, and the co-regulation of multiple members of the same pathway provide reasonable assurance that the data are reliable. We have established this level of confidence through multiple validation/correlation analyses over the past 20 years, both published (below) and unpublished. These correlation studies include comparisons with quantitative RT-PCR, RNASeq, Immunocytochemistry, and Luminex microsphere hybrid capture platform.

Correlation to RT-PCR:

Naciff, J.M., Jump, M.L., Torontali, S.M., Carr, G.J., Tiesman, J.P., Overmann, G.J., Daston, G.P. (2002) Gene Expression Profile Induced by 17α-Ethynyl Estradiol, Bisphenol A, and Genistein in the Developing Female Reproductive System of the Rat. Toxicological Sciences 68:184-199. PMID 12075121. Winner of “Best Publication of 2002” Award by The Society of Toxicology.

Naciff, J.M., Overmann, G.J., Torontali, S.M., Carr, G.J., Tiesman, J.P., Richardson, B.D., Daston, G.P. (2003) Gene Expression Profile Induced by 17-α-Ethynyl Estradiol in the Prepubertal Female Reproductive System of the Rat. Toxicological Sciences 72:314-330. PMID 12655037.

Naciff, J.M., Overmann, G.J., Torontali, S.M., Carr, G.J., Tiesman, J.P., Daston, G.P. (2004) Impact of the Phytoestrogen Content of Laboratory Animal Feed on the Gene Expression Profile of the Female Reproductive System of the Immature Rat. Environmental Health Perspectives 112:1519-1526. PMID 15531437.

Hu, T., Gibson, D.P., Carr, G.J., Torontali, S.M., Tiesman, J.P., Chaney, J.G., Aardema, M.J. (2004) Identification of a Gene Expression Profile That Discriminates Indirect-Acting Genotoxins From Direct-Acting Genotoxins. Mutation Research 549:5-27. PMID 15120960.

Naciff, J.M., Torontali, S.M., Overmann, G.I., Carr, G.J., Tiesman, J.P., Daston, G.P. (2005) Evaluation of the Gene Expression Changes Induced by 17-alpha-Ethynyl Estradiol in the Immature Uterus/Ovaries of the Rat Using High Density Oligonucleotide Arrays. Birth Defects Research Part B: Developmental and Reproductive Toxicology 74:164-184. PMID 15834898.

Naciff, J.M., Hess, K.A., Overmann, G.J., Torontali, S.M., Carr, G.J., Tiesman, J.P., Foertsch, L.M., Martinez, J.E., Daston, G.P. (2005) Gene Expression Changes in the Rat Testis by Transplacental Exposure to High and Low Doses of 17α-Ethynyl Estradiol, Genistein or Bisphenol A. Toxicological Sciences 86:396-416. PMID 15901920.

Hoffmann, J.L., Torontali, S.P., Thomason, R.G., Lee, D.M., Brill, J.L., Price, B.B., Carr, G.J., Versteeg D.J. (2006) Hepatic gene expression profiling using Genechips in zebrafish exposed to 17-ethynylestradiol. Aquatic Toxicology 79: 233–246

Naciff, J.M., Overmann, G.J., Torontali, S.M., Carr, G.J., Khambatta, Z.S., Tiesman, J.P., Richardson, B.D., Daston, G.P. (2007) Uterine Temporal Response to Acute Exposure to 17α-Ethynyl Estradiol in the Immature Rat. Toxicological Sciences 97:467-490. PMID 17351261.

MacSharry, J., O'Mahony, L., Fanning, A., Bairead, E., Sherlock, G., Tiesman, J., Fulmer, A., Kiely, B., Shanahan, F., Quigley, E.M.M. (2008) Mucosal cytokine imbalance in irritable bowel syndrome (IBS). Scandinavian Journal of Gastroenterology 43:1467-1476. PMID 18752146. >100 citations.

Naciff, J.M., Khambatta, Z.S., Thomason, R.G., Carr, G.J., Tiesman, J.P., Singleton, D.W., Khan, S.A., Daston, G.P. (2009) The Genomic Response of a Human Uterine Endometrial Adenocarcinoma Cell Line to 17α-Ethynyl Estradiol. Toxicological Sciences 107:40-55. PMID 18936297.

Naciff, J.M., Khambatta, Z.S., Reichling, T.D., Carr, G.J., Tiesman, J.P., Singleton, D.W., Khan, S.A., Daston, G.P. (2010) The Genomic Response of Ishikawa Cells to Bisphenol A Exposure is Dose- and Time-Dependent. Toxicology 270:137-149. PMID 20170705.

Hu, T., Khambatta, Z.S., Hayden, P.J., Bolmarcich, J., Binder, R.L., Robinson, M.K., Carr, G.J. Tiesman, J.P., Jarrold, B.B., Osborne, R.M., Reichling, T.D., Nemeth, S.T., Aardema, M.J. (2010) Xenobiotic Metabolism Gene Expression in the EpiDermTM In Vitro 3D Human Epidermis Model Compared to Human Skin. Toxicology In Vitro 24:1450-1463. PMID 20350595.

Naciff, J.M., Khambatta, Z.S., Carr, G.J., Tiesman, J.P., Singleton, D.W., Khan, S.A., Daston, G.P. (2016) Dose- and Time-Dependent Transcriptional Response of Ishikawa Cells Exposed to Genistein. Toxicological Sciences 151:71-87. PMID 26865667.

Correlation to Immunocytochemistry:

Bachelor, M., Binder, R.L., Cambron, T., Kaczinksy, J.R., Spruell, R., Wehmeyer, K.R., Reilman, R., Adams, R., Tiesman, J.P., Schnell, D.J., Wang, Y., Robinson, M.K., Mills, K., Bascom, C.C., Isfort, R.J., DiColandrea, T. (2014) Transcriptional Profiling of Epidermal Barrier Formation In Vitro. Journal of Dermatological Science 73:187-197. PMID 24314759.

Comprehensive correlation to hybrid capture on Fluorescent microspheres:

Naciff, J.M., Richardson, B.D., Oliver, K.G., Jump, M.L., Torontali, S.M., Juhlin, K.D., Carr, G.J., Paine, J.R., Tiesman, J.P., Daston, G.P. (2005) Design of a Microspheres-Based High-Throughput Gene Expression Assay to Determine Estrogenic Potential. Environmental Health Perspectives, 113:1164-1171. PMID 16140622.

Tiesman, J.P. and Torontali, S.M. (2007) Screening Gene Signatures: Strategies for Secondary Screening of Gene Expression Biomarkers. American Drug Discovery 2:6-15.

Torontali, S.M., Naciff, J.M., Juhlin, K.D., Tiesman, J.P. (2008) Profiling Gene Expression Signatures Using Fluorescent Microspheres. In: Biomarker Methods in Drug Discovery and Development (F. Wang ed.) Humana Press (ISBN: 978-1-934115-23-7).

Comprehensive correlation to RNASeq:

Tiesman, J.P., Schnell, D.J., Xu, J., Isfort, R.J., Kasibhatla, B. (2015). Complementarity of Microarray and RNA-Seq Data: A Case Study for Deep Understanding if Skin Aging. Genetic Engineering News. February 2015.
